# USP11-mediated LSH deubiquitination inhibits ferroptosis in colorectal cancer through epigenetic activation of CYP24A1

**DOI:** 10.1038/s41419-023-05915-9

**Published:** 2023-07-06

**Authors:** Junyi Duan, Daoyuan Huang, Cheng Liu, Yangbo Lv, Lei Zhang, Fen Chang, Xiangyu Zeng, Li Li, Weiping Wang, Genze Shao

**Affiliations:** 1 Department of Cell Biology, School of Basic Medical Sciences, Peking University Health Science Center, 100191 Beijing, China; 2 Advanced Innovation Center for Human Brain Protection, and National Clinical Research Center for Geriatric Disorders, Xuanwu Hospital Capital Medical University, 100053 Beijing, China; 3 Program for Cancer and Cell Biology, Department of Human Anatomy, Histology and Embryology, School of Basic Medical Sciences, Peking University International Cancer Institute, 100191 Beijing, China; 4 Colorectal Department of Quzhou Affiliated Hospital of Wenzhou Medical University, Quzhou People’s Hospital, 324000 Quzhou, China; 5 Department of Otorhinolaryngology, Qilu Hospital of Shandong University, NHC Key Laboratory of Otorhinolaryngology (Shandong University), 250012 Jinan, China; 6 Department of Biochemistry and Molecular Biology, Peking University Health Science Center, 100191 Beijing, China

**Keywords:** Ubiquitylation, Colorectal cancer

## Abstract

Ferroptosis is an iron-dependent form of regulated cell death characterized by lipid peroxidation. Colorectal cancer (CRC) cells evade ferroptosis despite their requirement of substantial iron and reactive oxygen species (ROS) to sustain active metabolism and extensive proliferation. However, the underlying mechanism is unclear. Herein, we report the role of lymphoid-specific helicase (LSH), a chromatin-remodeling protein, in suppressing erastin-induced ferroptosis in CRC cells. We demonstrate that erastin treatment leads to dose- and time-dependent downregulation of LSH in CRC cells, and depletion of LSH increases cell sensitivity to ferroptosis. Mechanistically, LSH interacts with and is stabilized by ubiquitin-specific protease 11 (USP11) via deubiquitination; this interaction was disrupted by erastin treatment, resulting in increased ubiquitination and LSH degradation. Moreover, we identified cytochrome P450 family 24 subfamily A member 1 (*CYP24A1*) as a transcriptional target of LSH. LSH binds to the *CYP24A1* promoter, promoting nucleosome eviction and reducing H3K27me3 occupancy, thus leading to transcription of *CYP24A1*. This cascade inhibits excessive intracellular Ca^2+^ influx, thereby reducing lipid peroxidation and ultimately conferring resistance to ferroptosis. Importantly, aberrant expression of USP11, LSH, and CYP24A1 is observed in CRC tissues and correlates with poor patient prognosis. Taken together, our study demonstrates the crucial role of the USP11/LSH/CYP24A1 signaling axis in inhibiting ferroptosis in CRC, highlighting its potential as a therapeutic target in CRC treatment.

## Introduction

Ferroptosis, a form of iron-dependent lipid peroxidation-regulated cell death (RCD), is governed by intricate signaling networks [1, 2]. Dysregulation of the cystine/glutamate antiporter (system xc-) and antioxidative systems can trigger ferroptosis. Mechanistically, carrier family 7 member 11 (SLC7A11) supplies cysteine for glutathione (GSH) synthesis, which is then utilized by GSH-dependent glutathione peroxidase 4 (GPX4) to inhibit ferroptosis through catalyzing lipid peroxide degradation [3]. Additionally, suppressor protein 1 (FSP1), dihydroorotate dehydrogenase (DHODH), and GTP cyclohydrolase 1 (GCH1) can inhibit lipid peroxidation in a GPX4/GSH-independent manner [4–6]. Metabolic disturbances, such as an imbalance of intracellular iron pool, and unsaturated fatty acid synthesis and catabolism can also induce ferroptosis [7]. Ferroptosis is implicated in various physiological processes (e.g., antiviral immunity, aging) and pathological conditions (e.g., degenerative diseases, ischemia-reperfusion injury, cancer) [7, 8]. Malignant cells with characteristics such as resistance to chemotherapy or radiotherapy, poor differentiation, or high metastatic potential tend to be more susceptible to ferroptosis due to their metabolic reprogramming [9], indicating an unusual vulnerability exploited for targeted therapeutic opportunities. Clinical trials have demonstrated the potential efficacy of inducing ferroptosis in certain cancers, and induction of ferroptosis combined with other conventional cancer therapies has also demonstrated promising synergistic effects in preclinical cancer models [10–12]. Interestingly, colorectal cancer (CRC) cells require significant iron and reactive oxygen species (ROS) to sustain their active metabolism and proliferation [13, 14]. However, they exhibit resistance to ferroptosis, suggesting CRC cells have evolved sophisticated strategies to evade this form of cell death. Nevertheless, the underlying mechanisms of ferroptosis evasion in CRC cells remain poorly understood.

Lymphoid-specific helicase (LSH) belongs to the sucrose non-fermentable 2 family of ATP-dependent chromatin remodelers [15], and is involved in DNA methylation, nucleosome remodeling, histone modifications, heterochromatin formation, and DNA damage repair [16]. Through epigenetic modulation of chromatin structure and accessibility, LSH regulates the transcription of genes critical for stem cell pluripotency, lymphocyte development, immune regulation, and DNA replication [16–20]. LSH has recently gained attention for its regulation of ferroptosis-associated genes that play a crucial role in lipid peroxidation, ROS accumulation, and iron pool imbalance, which are key inducers of ferroptosis. For instance, LSH promotes *SLC7A11* transcription to facilitate cystine uptake, thereby inhibiting ferroptosis in leukemia [21]. Another repressive role of LSH in ferroptosis is to recruit WDR76 to the promoters of *SCD1* and *FADS2*, the upregulation of which could further increase GPX4 expression, elevate GSH/GSSG ratio, reduce ROS levels, and decrease iron concentration [22]. Additionally, LSH upregulates the expression of cystathionine-β-synthase (CBS), a vital enzyme in the transsulfuration pathway of cysteine synthesis, thus enhancing cellular antioxidant capacity against ferroptosis [23]. This ferroptosis-suppressive role of LSH is mediated through the LSH/EAVL1-mediated upregulation of LINC00336, a long noncoding RNA that competitively binds to *CBS* mRNA with miR-6852, preventing *CBS* degradation [23]. LSH also suppresses the expression of P53RRA, a long noncoding RNA that promotes ferroptosis in a p53-dependent manner in lung cancer [24]. However, the precise mechanism by which LSH regulates ferroptosis under different physiological and pathological conditions, especially CRC, remains largely unexplored. Moreover, knowledge regarding the regulatory mechanism of LSH protein stability during ferroptosis is limited.

Here we reported the role and underlying mechanism of LSH in the suppression of ferroptosis in CRC. We identified the USP11/LSH/CYP24A1 axis as an important pathway that inhibits ferroptosis.

## Materials and methods

### Plasmid construction

The coding sequences of USP11, LSH, and CYP24A1 were amplified from the GES-1 cDNA and cloned into different vectors to generate plasmids with various tags. Truncations containing the CYP24A1 promoter sequence were amplified from HCT116 genomic DNA. Ub mutant (K11R, K48R, K11 & K48R) and USP11 mutant (C318A) plasmids were obtained using the Fast Mutagenesis System (FM111-01, TransGen). The primer sequences are provided in Supplementary Tables S1–S3.

### Cell culture and reagents

Cell lines derived from Prof. Shao and Prof. Tong lab were used. All cell lines were authenticated and cultured according to ATCC standards. Reagents used in cell culture were as follows: Fetal bovine serum (SH30406.05, HyClone), penicillin/streptomycin (15140148, Thermo), erastin (S7242, Selleck), ferrostatin-1 (S7243, Selleck), vitamin D3 (S4063, Selleck), mitoxantrone (S1210, Selleck), MG132 (S2619, Selleck), puromycin (S7417, Selleck), cycloheximide (S7418, Selleck), bafilomycin A1 (S1413, Selleck), ketoconazole (HY-B0105, MCE), and CoCl_2_ (232696, Sigma-Aldrich).

### Stable cell lines construction

*LSH*, *USP11*, and *CYP24A1* knockout cell lines (LSH-KO, USP11-KO, CYP24A1-KO), knockdown cell lines (shLSH, shUSP11, shCYP24A1), and stably overexpressing cell lines were generated using the psPAX2 and pMD2.G lentiviral packaging systems. Briefly, a certain proportion of lentiviral plasmids were transferred into HEK293T cells, after 72 h, the viral supernatant was harvested to infect target cells at 50% confluence with 5 μg/ml polybrene (40804ES76, Yeasen). After 48 h, puromycin was added to screen positive cell pools or clones.

### Liquid chromatography-tandem mass spectrometry

Immunoprecipitated proteins were separated by SDS-PAGE gel, stained with Coomassie blue and decolorized. Cut off the protein bands on the gel and use the NanoLC-Q EXACTIVE Mass Spectrometer (Thermo Fisher Scientific) for further mass spectrometry analysis.

### RNAi and transfection

siRNAs were purchased from GenePharma (Suzhou, China), and Lipofectamine RNAiMAX Reagent (13778-150, Invitrogen) was used for transfection. Briefly, 30% confluent cells were transfected with siRNA. After 48 h, replaced the medium and harvested the cells at 72 h. The target siRNA sequences are provided in Supplementary Table S4.

### Western blot

Cells were lysed in RIPA buffer (50 mM Tris-HCl at pH 7.4, 150 mM NaCl, 1% NP-40, 1% sodium deoxycholate, 0.1% SDS) with cocktail (protease inhibitor). The protein supernatant was collected by centrifugation and the concentration was quantified. The proteins were transferred onto the PVDF (GE) membrane by electrophoresis and electrical transfer. Membranes were blocked with 5% non-fat milk and incubated with primary antibodies overnight at 4°C. On the second day, membranes were incubated with secondary antibodies for 1 h at RT. Images were obtained by X-ray film exposure. The antibodies used for western blot (WB) analysis were USP11 (Abcam, ab109232), LSH (Sigma, HPA063242), CYP24A1 (Sigma, HPA022261), GPX4 (Abcam, ab125066), SLC7A11 (CST, 12691), Multi-Ub (MBL, D058-3), Flag-tag (Sigma, F3165), HA-tag (CST, 3724), MYC-tag (MBL, M192-3S), His-tag (MBL, D291-3S), H3K27me3 (Abcam, ab192915), β-actin (Sigma, 4930), Tubulin (Sigma, T9026), H3 (CST, 60932), and horseradish peroxidase (HRP)-conjugated secondary antibodies (Jackson, 111-035-003; 115-035-003).

### Co-immunoprecipitation

Cells were lysed in IP lysis buffer (50 mM Tris-HCl at pH 7.4, 150 mM NaCl, 1 mM EDTA, 0.3% NP-40) with cocktail, sonicated, and then centrifuged. The protein supernatant was incubated with 2 μg antibodies and 20 μL protein A/G agarose (Santa Cruz, sc-2003) at 4 °C for 6 h. The immunoprecipitates were washed, eluted, and analyzed by WB.

### Protein expression and purification

Flag-tagged USP11 proteins were obtained in vitro using a TNT® T7 Quick Coupled Transcription/Translation System (Promega, L1171). GST-tagged proteins were obtained from *E. coli*. Briefly, bacteria were induced with 0.5 mM isopropyl-d-1-thiogalactopyranoside (IPTG) at 30 °C for 6 h, sonicated in cold PBS with 1 mM phenylmethylsulfonyl fluoride (PMSF). The protein supernatant obtained by centrifugation was incubated with 50 μL Glutathione Sepharose 4B resins (YEASEN, 20507ES10) at 4 °C overnight. The immobilized proteins on the resins were washed and eluted using elution buffer (50 mM Tris-HCl, pH 8.0, 10 mM reduced glutathione, 1 mM DTT). To prepare ubiquitinated LSH, HEK293T cells were transfected with Flag-LSH and HA-Ub, and lysed for co-immunoprecipitation (Co-IP). HA beads were used for the initial purification, and the eluted proteins were further purified with Flag M2 beads.

### GST pull-down assay

The GST, GST-LSH, and its truncations bound to Glutathione Sepharose 4B resins were incubated with in vitro translated Flag-tagged USP11 in binding buffer (50 mM Tris-HCl pH 7.4, 150 mM NaCl, 0.2% Triton X-100, 10% glycerol, 1 mM DTT, cocktail) at 4 °C overnight, respectively. Subsequently, the resins were washed and eluted for further WB analysis.

### In vivo ubiquitination

Cells were lysed in denaturing buffer (8 M urea, 20 mM Tris-HCl pH 8.0, 200 mM NaCl, 0.05% NP-40, 15 mM imidazole) and sonicated. The cell extracts were incubated with Ni-NTA beads (QIAGEN; 30230) at 4 °C for 6 h. Washed the beads and added the same volume of denaturing buffer containing 2× SDS-loading buffer and boiled for 10 min, and the supernatant was analyzed by WB.

### In vitro deubiquitination

Flag-LSH conjugated with HA-Ub was reacted with GST-USP11 or GST-USP11 mutant (C318A) in deubiquitination buffer (50 mM Tris-HCl, pH 7.5, 100 mM NaCl, 5 mM MgCl_2_, 10 mM DTT, 5% glycerin) at 37 °C for 2 h. After centrifugation, the supernatant was used for WB detection.

### Immunofluorescence

Cells seeded on coverslips at a suitable confluence were fixed in 4% paraformaldehyde for 15 min, washed, permeabilized with 0.5% Triton X-100 for 10 min. After blocking with 5% goat serum, cells were incubated with primary antibodies at 4 °C overnight. The next day, cells were washed and incubated with secondary antibodies and Hoechst 33342 (Invitrogen) for 1 h at RT. The coverslips were mounted and images were captured using a Zeiss Axiolmager M2 microscope. Antibodies were USP11 (Proteintech, 22340-AP), LSH (Santa Cruz, sc-46665), fluorescent secondary antibodies (Jackson, 111-585-003; 115-545-003).

### RNA extraction, RT-qPCR, and RNA-sequencing

RNA was extracted and converted to cDNA using a Reverse Transcription Kit (MF011-T, mei5bio). Quantitative PCR (qPCR) was performed on an Applied Biosystems 7500 Real-Time PCR System using SYBR Green qPCR Mix (Selleck, B21703). The primers used for qPCR analysis are provided in Supplementary Table S5. Whole transcriptome sequencing was performed at the Beijing Genome Institute (BGI).

### GPx activity, GSH, and GSSG measurement

Glutathione peroxidase (GPx) activity was tested using a Glutathione Peroxidase Test Kit (Beyotime, S0056). Glutathione (GSH) and glutathione disulfide (GSSG) were measured using a Glutathione Peroxidase Test Kit (Beyotime, S0053).

### Lipid peroxidation detection

Cells were harvested, washed, and incubated with 5 µM BODIPY 581/591 C11 dye (Invitrogen, D3861) in PBS at 37 °C for 0.5 h. Subsequently, cells were washed and resuspended in PBS. A total of 20,000 cells per sample were analyzed for lipid peroxidation levels using the FL1 channel of a Becton Dickinson FACS-Calibur2 machine. Images were acquired using Flowjo10.8.1 software.

### Intracellular Fe^2+^detection

Cells were seeded in 35 mm glass-bottom culture dishes (EpiZyme, BDD012035), washed with HBSS buffer, and incubated with 1 μM FerroOrange (DOJINDO, F374) in HBSS at 37°C in a containing a 5% CO_2_ incubator for 0.5 h. Afterward, cells were stained with Hoechst 33342 (Invitrogen) for 5 min at RT. Live cell images were captured using a Nikon A1-R microscope.

### Intracellular Ca^2+^ detection

Cells were collected, washed, and incubated with 2.5 μM Fluo-4 AM (F14201, Thermo) in HBSS at 37 °C for 40 min in the dark. Then cells were resuspended in HBSS containing 1% FBS at 37 °C for 10 min. A total of 20,000 cells per sample were analyzed for Ca^2+^ levels using the FL1 channel of a Becton Dickinson FACS-Calibur2 machine. Images were acquired using Flowjo10.8.1 software.

### Cell viability and proliferation detection

In all, 3000 cells were seeded in each well of 96-well plate. After treatment with the indicated drugs, cells were incubated with fresh medium containing 10% Cell Counting Kit-8 (CCK8) reagent (DOJINDO, CK04) at 37 °C for 2 h, and the absorbance was measured at 450 nm using a FLUO star Omega microplate reader (BMG Labtech).

### Colony formation assay

In all, 1000 cells/well were seeded in six-well plate. Cells were treated with the indicated drugs for 24 h and then cultured in fresh medium for 14 days. Colonies were fixed with 4% paraformaldehyde for 20 min, stained with 0.5% crystal violet (E607309-0100) for 5 min, and washed with clean water.

### Luciferase reporter assay

The dual-luciferase reporter assay was performed according to the instructions of the kit (Promega, E1910). Briefly, the appropriate proportion of plasmids were transferred into HEK293 cells, then the cells were washed and lysed at 4 °C for 10 min. The supernatant of the lysate was transferred to 96-well plates and incubated with firefly luciferase. Each sample was analyzed in triplicate. Luciferase activity was assayed using a Gen5 microplate reader (BIOTEK) and Renilla luciferase was used as an internal reference.

### ChIP assay

Cells were crosslinked with 1% formaldehyde for 15 min at RT and neutralized with glycine to a final concentration of 125 mM. Then, cells were washed, lysed in lysis buffer (50 mM Tris-HCl pH 8.0, 5 mM EDTA, 1% SDS), and sonicated. After centrifugation, the supernatant was diluted in dilution buffer (20 mM Tris-HCl pH 8.0, 150 mM NaCl, 2 mM EDTA, 1% Triton X-100) and incubated with 2 μg ChIP-grade antibodies at 4 °C overnight. The following day, 20 μL Protein A/G agarose (Santa Cruz, sc-2003) were added and incubated for an additional 3 h at 4 °C. Beads were washed and eluted with 100 µL elution buffer (1% SDS, 100 mM NaHCO_3_). The eluent was incubated in a water bath at 60 °C for 8 h, followed by additional incubation with 20 µL proteinase K (NEB, P8107S) and 20 µL RNaseA (NEB, T3018L) at 45 °C for 1 h. DNA was purified using a PCR purification kit (QIAGEN, 28106) and analyzed by qPCR. The primer sequences are provided in Supplementary Table S5. Antibodies used were LSH (Abcam, ab3851), H3 (Abcam, ab1791), H3K4me3 (Abcam, ab8580), H3K9me3 (Abcam, ab8898), H3K27me3 (Abcam, ab192985), H3K36me3 (Abcam, ab9050), H3K18ac (Abcam, ab1191), H3K27ac (Abcam, ab4729), normal IgG (CST, 2729; 61656).

### Micrococcal nuclease mapping assay

The micrococcal nuclease (MNase) mapping assay was performed as previously described [25, 26]. Briefly, cells were harvested, washed with PBS, and resuspended in hypertonic buffer A (300 mM sucrose, 2 mM Mg-acetate, 3 mM CaCl_2_, 10 mM Tris (pH 8.0), 0.1% Triton X-100, and 0.5 mM DTT) at a concentration of 1 × 10^8^ cells/ml. The suspension was incubated on ice for 5 min, followed dounced with a 2 ml dounce homogenizer 20 times. Nuclei were collected by centrifuging at 720 g for 5 min at 4 °C, washed twice with buffer A, and resuspended in buffer D (25% glycerol, 5 mM Mg-acetate, 50 mM Tris (pH 8.0), 0.1 mM EDTA, 5 mM DTT). The nuclei were collected, washed twice with buffer D, and resuspended in buffer MN (60 mM KCl, 15 mM NaCl, 15 mM Tris (pH 7.4), 0.5 mM DTT, 0.25 M sucrose, 1.0 mM CaCl_2_). The nuclei were incubated with 0, 1, 10, and 100 units of MNase (NEB, M0247S) per reaction at 37 °C for 15 min. The reactions were then terminated with by adding EDTA and SDS to final concentrations of 12.5 mM and 0.5%, respectively. DNA was visualized by electrophoresis with a 2% agarose gel, and the mono-nucleosome-sized DNA fragments were extracted by QIAquick Gel Extraction Kit (QIAGEN, 28704) for subsequent quantitative PCR analysis. The Primers used in this assay are provided in Supplementary Table S6.

### Transmission electron microscopy

Samples were prepared as described previously [27]. Subsequently, the samples were observed using a Spirit transmission electron microscope (FEI Company) operating at 100 kV. Digital images were captured using an AMT imaging system at a high-resolution electron microscopy facility.

### In vivo xenograft

In all, 24 5-week-old female athymic BALB/c nude mice with comparable body weight were randomly assigned to 4 groups, with 6 mice per group. Each mouse was subcutaneously injected with a 100 µL tumor cell dilution containing 1 × 10^6^ cells into the right flank. Tumor growth was monitored and measured using calipers for a duration of 20 days post-injection, with tumor volume calculated using the formula: *V* = length × width^2^ × 1/2. Mouse weight was recorded every 3 days until the study endpoint.

### Immunohistochemistry

Tissues were fixed in formalin, embedded in paraffin, and sliced into thin sections. Samples were dewaxed, rehydrated, and then antigen repaired using 0.1 M EDTA (pH 9.0) at 100 °C for 20 min. Subsequently, samples were treated with 3% H_2_O_2_ in the dark for 10 min. Next, the samples were blocked with 5% goat serum at RT for 1 h and incubated with primary antibodies at 4 °C overnight. On the second day, the samples were washed, incubated with HRP-conjugated secondary antibodies for 1 h at RT, and stained with DAB (ZSGB-BIO, ZLI-9018). Finally, the samples were dehydrated using increasing concentrations of ethanol. Antibodies were USP11 (Proteintech, 22340-AP), LSH (Sigma, HPA063242), CYP24A1 (Sigma, HPA022261), 4-HNE (Abcam, ab46545), and Ki-67 (Abcam, ab15580).

### Statistical analysis

Statistical data are presented as mean ± SD. Student’s *t* test (two-tailed), one-way analysis of variance (ANOVA) with Tukey’s test, *χ*^2^ test, and Linear regression analysis were performed using GraphPad Prism software (version 8.0). RNA-seq differential gene analysis, Pearson’ correlation, and Fisher’ exact test were performed using R software. *P* > 0.05 (ns: non-significant); *P* < 0.05 was considered statistically significant; *P < 0.05, **P < 0.01, ***P < 0.001, and ****P < 0.0001.

## Results

### LSH is aberrantly expressed in CRC

**Fig. 1:**
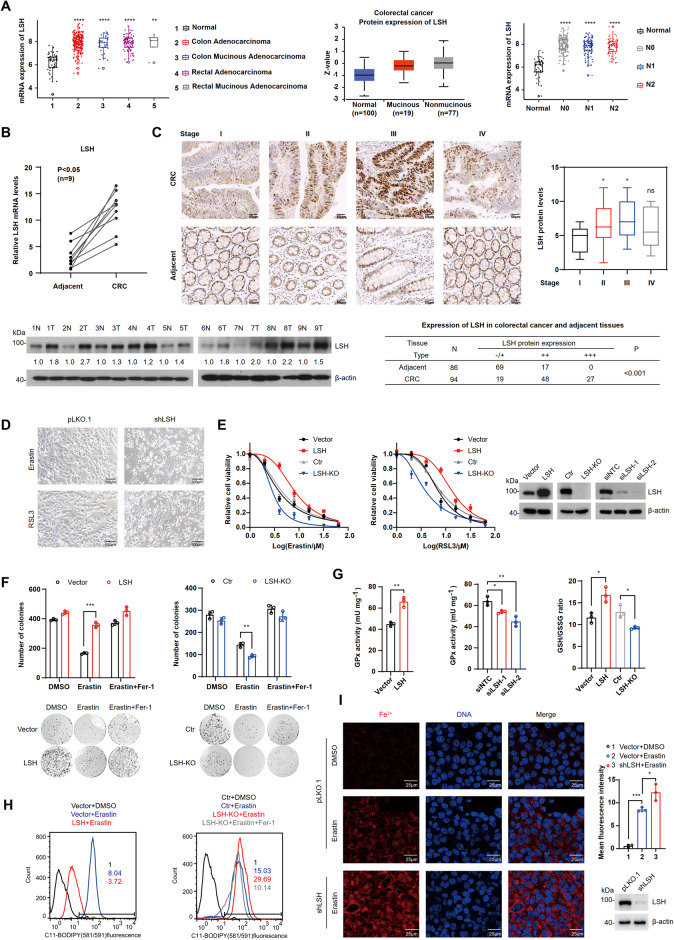
Upregulation of LSH expression inhibits ferroptosis in CRC. **A** Analysis of LSH expression in CRC and normal tissues from TCGA and CPTAC databases (Nx represents the extent of CRC lymph node metastasis). **B** RT-qPCR (top) and WB (bottom) analyses of LSH expression levels in paired CRC samples. N, adjacent normal tissues; T, CRC tumor tissue (*n* = 9). **C** Immunohistochemical analysis of LSH expression in paired CRC samples at different clinical stages. Scale bar: 50 μm. Statistical results are included (right). The *P*-value was calculated using the χ^2^ test (bottom). **D** Representative images showing cell death. SW620 cells were treated with erastin (20 μM) or RSL3 (20 μM) for 24 h. Scale bar, 500 μm. **E** HCT116 cells overexpressing or depleted of LSH were treated with different concentrations of erastin or RSL3 for 24 h, and cell viability was assayed. Values are presented as mean ± SD (*n* = 3). **F** Clonal expansion analysis of HCT116 cells overexpressing or depleted of LSH. The cells were incubated with erastin (20 µM) for 24 h or Fer-1 (5 µM) for 20 h. Values are presented as mean ± SD (*n* = 3). **G** Intracellular GPx activity and GSH and GSSG levels were measured in LSH overexpressing or LSH-deficient HCT116 cells. Values are presented as mean ± SD (*n* = 3). **H** Analysis of lipid peroxidation in LSH overexpressing or LSH-deficient cells treated with erastin (20 μM) for 24 h or Fer-1 (5 μM) for 20 h. **I** Intracellular Fe^2+^ levels were examined in cells incubated with DMSO or erastin (20 µM) for 24 h using laser scanning confocal microscopy with FerrOrange staining. Scale bar, 25 µm.

To investigate the potential association between LSH and CRC, we performed bioinformatics analysis using the TCGA and CPTAC databases. We found that LSH was highly expressed and positively correlated with lymph node metastasis in CRC (Fig. 1A). We further examined LSH expression in clinical CRC patients and observed higher levels of LSH in cancer tissues compared to adjacent tissues, both at the mRNA and protein levels. (Fig. 1B). Moreover, we found overexpression of LSH was positively correlated with early-to-mid clinical staging, particularly in stages I-III of CRC (Fig. 1C). These results suggest that LSH overexpression may play an important role in CRC tumorigenesis and development.

### LSH is essential to inhibit erastin-induced ferroptosis in CRC cells

To investigate whether upregulated LSH is associated with ferroptosis evasion in CRC cells, we induced ferroptosis in CRC cells using two ferroptosis activators, erastin and RSL3. The results showed that LSH knockdown or LSH knockout (LSH-KO, Fig. S1B) resulted in substantial cell death when treated with erastin or RSL3, compared to the control (Fig. 1D, E). Conversely, cells overexpressing LSH exhibited increased resistance to these drugs, particularly erastin (Fig. 1E). These results were further confirmed through colony formation assays (Fig. 1F). Notably, erastin-induced ferroptosis was significantly alleviated by treatment with an iron-chelating agent ferrostatin-1 (Fer-1) 2 (Fig. 1F). Glutathione peroxidase (GPx) plays an antioxidant role in ferroptosis by converting lipid peroxides to lipid alcohols through reduced GSH, thereby protecting cells from membrane oxidation damage [28]. We observed that LSH expression positively correlated with intracellular GPx activity and GSH generation, while LSH-deficient cells exhibited the opposite effect (Fig. 1G). Since an increase in lipid peroxides is another crucial signal for ferroptosis [29], the fluorescent probe-C11-BODIPY was used to measure lipid peroxidation levels. We found that LSH overexpression repressed lipid peroxidation, while LSH depletion increased it, which could be rescued by Fer-1 treatment (Fig. 1H). Excess iron triggers ferroptosis by generating hydroxyl radicals through the iron-dependent Fenton reaction and activating iron-containing lipoxygenase, resulting in ROS accumulation and lipid peroxidation [30, 31]. Accordingly, we observed significantly higher Fe^2+^ levels in LSH-deficient cells compared to control cells after erastin treatment (Fig. 1I). Taken together, these findings indicate that LSH inhibits erastin-induced ferroptosis in CRC cells.

### LSH is destabilized during erastin-induced ferroptosis

**Fig. 2:**
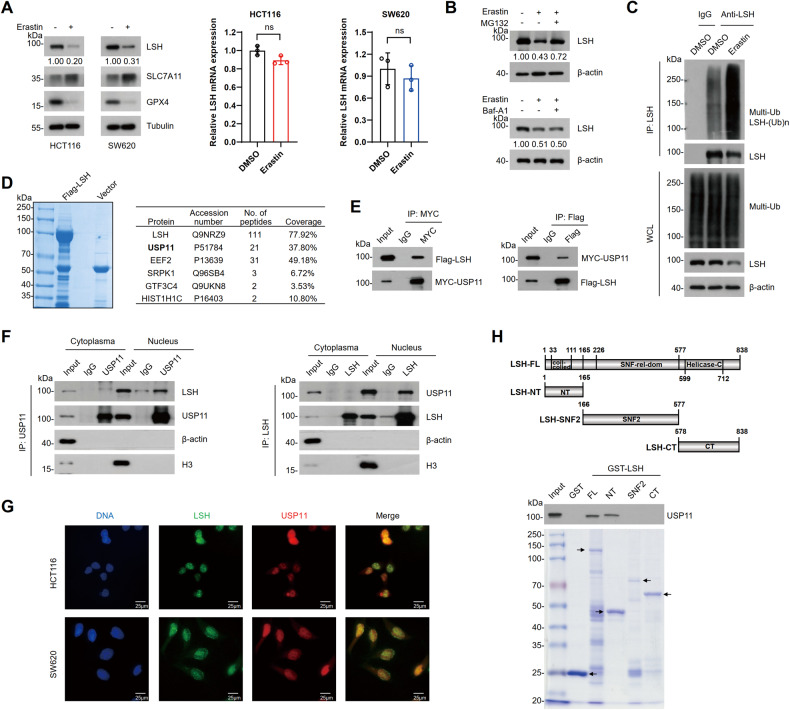
LSH is degraded upon erastin treatment and interacts with USP11. **A** Protein and mRNA expression of LSH were analyzed by WB (left) or RT-qPCR (right) in HCT116 and SW620 cells that treated with erastin (20 μM) for 24 h. Values are presented as mean ± SD (*n* = 3). **B** WB analysis of HCT116 cells that were treated with erastin (20 µM) for 16 h, then were incubated with MG132 (20 μM) for 6 h or Baf-A1 (1 µM) for 16 h. **C** HCT116 cells incubated with DMSO or erastin (20 µM) for 24 h were subjected to Co-IP with control IgG or LSH antibodies. Ubiquitination of LSH was analyzed by WB. **D** Liquid chromatography-tandem mass spectrometry analysis of Flag-LSH immunoprecipitated proteins from HCT116 cells (left). Representative peptide fragment counts and peptide coverage of indicated proteins are shown (right). **E** Co-IP analysis of the interaction between Flag-LSH and MYC-USP11 in HEK293T cells. MYC-IP (left); Flag-IP (right). **F** Cytoplasmic and nuclear extracts from HCT116 cells were fractionated for Co-IP using USP11 or LSH antibody. USP11-IP (left); LSH-IP (right). **G** Immunofluorescence analysis of USP11 and LSH in HCT116 and SW620 cells. Scale bar, 25 μm. **H** Mapping of the region in LSH that interacts with USP11. A schematic diagram of the constructed LSH truncations (top) and purified proteins with Coomassie blue staining (bottom) are shown.

Notably, when ferroptosis was induced by erastin in CRC cells, as indicated by an increase in SLC7A11 expression levels and a decrease in GPX4 expression levels, we observed a sharp decrease in LSH protein levels (Fig. 2A). Considering that downregulation of LSH may be achieved through erastin-induced transcriptional repression, we performed an RT-qPCR to test this hypothesis. Surprisingly, the levels of LSH mRNA were not significantly affected by these processes (Fig. 2A). As erastin-induced reduction of LSH could be restored by treatment with MG132 but not bafilomycin A1 (Fig. 2B), we presumed that LSH is degraded via the ubiquitin-proteasome pathway in erastin-induced ferroptosis. Supporting this assumption, increased ubiquitination of LSH was observed following erastin treatment (Fig. 2C). Collectively, these results suggest that erastin-induced destabilization of LSH during ferroptosis is related to ubiquitin-proteasome system (UPS).

### LSH associates with USP11

To investigate the regulation mechanism of LSH, liquid chromatography-tandem mass spectrometry was conducted. USP11, a cysteine protease of the ubiquitin-specific protease family (USPs) [32], was identified as an LSH-interacting protein (Fig. 2D). Additionally, histone cluster 1 H1 family member c (HIST1H1C), a previously known LSH-interacting protein [33] was also identified (Fig. 2D). Moreover, Co-IP experiments confirmed an obvious interaction between LSH and USP11, primarily within the nuclei of HCT116 cells (Fig. 2E, F). Consistently, immunofluorescence staining further demonstrated colocalization of endogenous LSH and USP11 within the nuclei of HCT116 or SW620 cells (Fig. 2G). Furthermore, the GST pull-down assay showed that LSH directly interacts with USP11 through its amino terminus (Fig. 2H).

### USP11 stabilizes LSH by deubiquitination

**Fig. 3:**
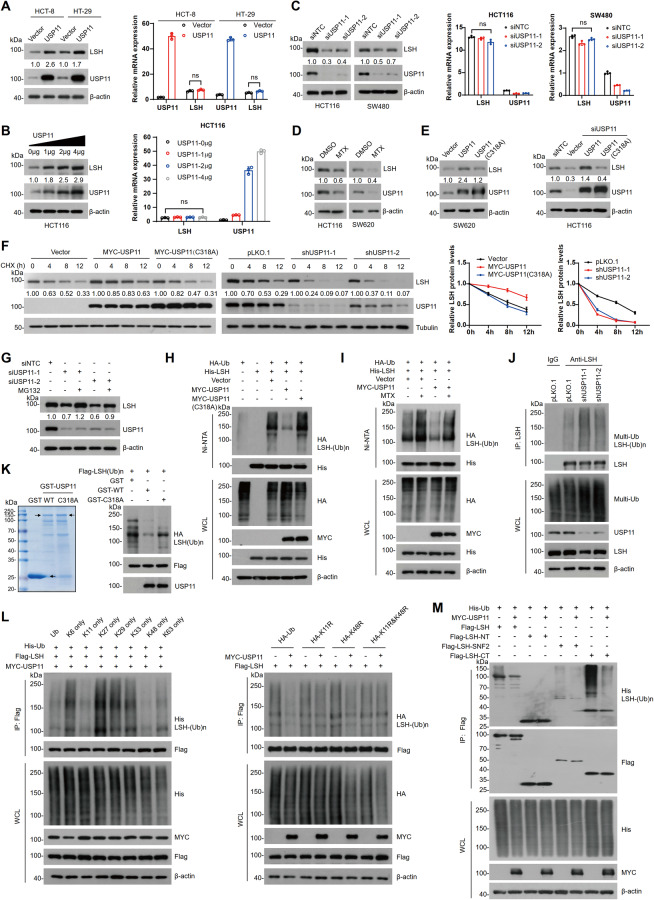
USP11 stabilizes LSH by deubiquitination. **A** HCT-8 and HT-29 cells transfected with control or USP11 plasmids were lysed for WB (left) and RT-qPCR (right) analyses. Values are presented as mean ± SD (*n* = 3). **B** HCT116 cells transfected with different doses of USP11 plasmids were collected for WB (left) and RT-qPCR (right) analyses. Values are presented as mean ± SD (*n* = 3). **C** SW480 and HCT116 cells transfected with control or USP11 siRNAs were collected for WB (left) or RT-qPCR analyses (right). Values are presented as mean ± SD (*n* = 3). **D** HCT116 and SW620 cells treated with DMSO or MTX (1 μM) for 12 h were lysed for WB analysis. **E** Cells transfected with control, USP11, or USP11 mutant (C318A) plasmids were lysed for WB analysis. **F** Cells were treated with cycloheximide (CHX, 50 μg/ml) for the indicated times and harvested for WB analysis with the indicated antibodies. Quantification was performed and normalized to tubulin levels. Values are presented as mean ± SD (*n* = 3). **G** HCT116 cells transfected with control or USP11 siRNAs were treated with DMSO or MG132 (20 μM) for 6 h and lysed for WB analysis. **H** HEK293T cells transfected with the indicated plasmids were incubated with MG132 (20 µM) for 6 h. Cells were lysed for the Ni-NTA pull-down assay, and ubiquitination of LSH was analyzed by WB. **I** HEK293T cells transfected with the indicated plasmids were treated with DMSO or MTX (1 μM) for 12 h and MG132 (20 µM) for 6 h. Cell lysates were analyzed as described above. **J** HCT116 cells were lysed for Co-IP with IgG or LSH antibodies, and ubiquitination of LSH was analyzed by WB. **K** In vitro deubiquitination (right). Purified GST, GST-USP11, and GST-USP11 (C318A) are shown (left). **L**, **M** HEK293T cells transfected with the indicated plasmids were incubated with MG132 (20 µM) for 6 h. Cells were lysed for Flag-IP and ubiquitination of LSH was analyzed by WB.

To determine the role of USP11 in regulating LSH stability in CRC cells, USP11 was overexpressed or knocked down in HCT-8, HT-29, or HCT116 cells, and LSH protein levels were examined. It was observed that overexpression of USP11 resulted in a significant increase in LSH protein levels (Fig. 3A, B), while USP11 knockdown had the opposite effect, with no notable change in LSH mRNA levels (Fig. 3A–C). As USP11 is a deubiquitinase (DUB), we tested whether its DUB activity is required for stabilizing LSH. Treatment of HCT116 and SW620 cells with mitoxantrone (MTX), a USP11 enzyme activity inhibitor [34], led to a pronounced reduction in LSH protein levels (Fig. 3D). Consistently, overexpression of wild-type USP11 (WT) but not the catalytically inactive mutant (C318A) increased LSH protein levels (Fig. 3E). The cycloheximide (CHX) chase assay further confirmed that overexpression of USP11, but not the C318A mutant, significantly enhanced LSH stability, whereas USP11 knockdown had the opposite effect (Fig. 3F). These results indicate that the DUB activity of USP11 is essential for regulating LSH stability.

To further confirm that USP11 stabilizes LSH through deubiquitination, USP11-deficient HCT116 cells were treated with MG132, and the LSH protein levels were examined. We found that USP11 depletion caused a remarkable decrease in LSH, which was rescued by MG132 treatment, suggesting that USP11-mediated stabilization of LSH is associated with the ubiquitin-proteasome pathway (Fig. 3G). In vivo ubiquitination experiments indicated that LSH ubiquitination levels were markedly decreased in the presence of WT USP11, but not its mutant (C318A) (Fig. 3H). Similarly, USP11-mediated decrease in LSH ubiquitination was restored by MTX treatment (Fig. 3I). Furthermore, the ubiquitination levels of endogenous LSH were increased upon USP11 depletion (Fig. 3J); and USP11 was capable of directly deubiquitinating LSH in vitro instead of its C318A mutant (Fig. 3K). Moreover, we also showed that USP11 primarily removes K11 and K48 ubiquitin chains, located at the C-terminus of LSH (Fig. 3L, M). These findings confirm that USP11 stabilizes LSH by deubiquitination.

### USP11 alleviates erastin-mediated degradation of LSH

**Fig. 4:**
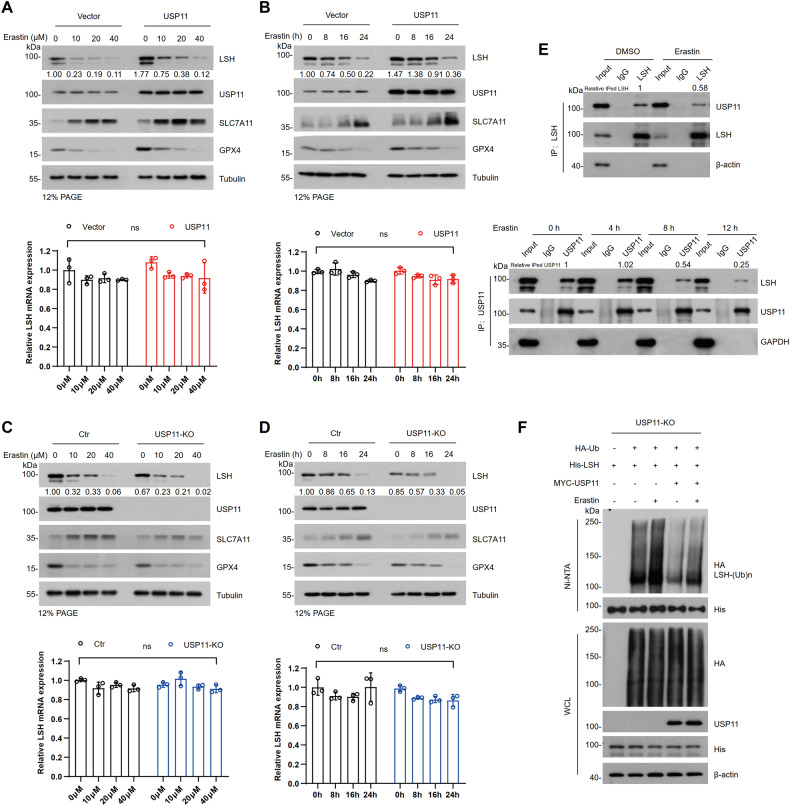
USP11 alleviates erastin-mediated degradation of LSH. **A**, **C** WB (top) and RT-qPCR (bottom) were analyzed in USP11 overexpressing stable cell line or USP11-KO cells treated with erastin for 24 h at the indicated concentrations. Values are presented as mean ± SD (*n* = 3). **B**, **D** WB (top) and RT-qPCR (bottom) were analyzed in USP11 overexpressing stable cell line or USP11- KO cells treated with erastin (20 µM) at the indicated time. Values are presented as mean ± SD (*n* = 3). **E** Co-IP with LSH antibody in HCT116 cells treated with DMSO or erastin (20 µM) for 24 h (top) and with USP11 antibody in HCT116 cells treated with erastin (20 µM) for 0, 4, 8, and 12 h respectively (bottom). **F** USP11-KO cells transfected with the indicated plasmids were incubated with DMSO or erastin (20 µM) for 24 h and MG132 (20 µM) for 6 h. Cell lysates were collected for Ni-NTA pull-down, and ubiquitination of LSH was analyzed by WB.

To investigate the involvement of USP11 in erastin-induced degradation of LSH, we examined the LSH protein levels in HCT116 cells treated with erastin. As shown in Fig. 4A–D, LSH protein levels gradually decreased over time or in a dose-dependent manner upon erastin treatment. Concurrently, there was an increase in SLC7A11 expression and a decrease in GPX4 expression, while the expression levels of USP11 remained unchanged. Importantly, forced expression of USP11 remarkably mitigated erastin-induced degradation of LSH, whereas USP11 knockout (USP11-KO, Figure S1A) accelerated LSH protein destabilization (Fig. 4A–D). Consistent with previous observations, there was minimal change in LSH mRNA levels (Fig. 4A–D). Additionally, LSH was found to have reduced interaction with USP11 in cells treated with erastin (Fig. 4E). The interaction between USP11 and LSH decreased with the duration of erastin treatment, and the reduction in interaction was significant at 8 hours when the level of LSH was not significantly affected (Fig. 4E). These results suggest that erastin disrupts the interaction between LSH and USP11, leading to increased ubiquitination and subsequent degradation of LSH. To confirm this assumption, we performed Co-IP using USP11-KO cells or USP11-KO cells that were reconstituted with USP11, and compared the ubiquitination levels of LSH under erastin treatment. The ubiquitination levels of LSH were elevated in the USP11-KO cells but recovered in the USP11-reconstituted KO cells (Fig. 4F). These findings support that USP11 alleviates erastin-induced degradation of LSH.

### *CYP24A1* is a transcriptional target of LSH

**Fig. 5:**
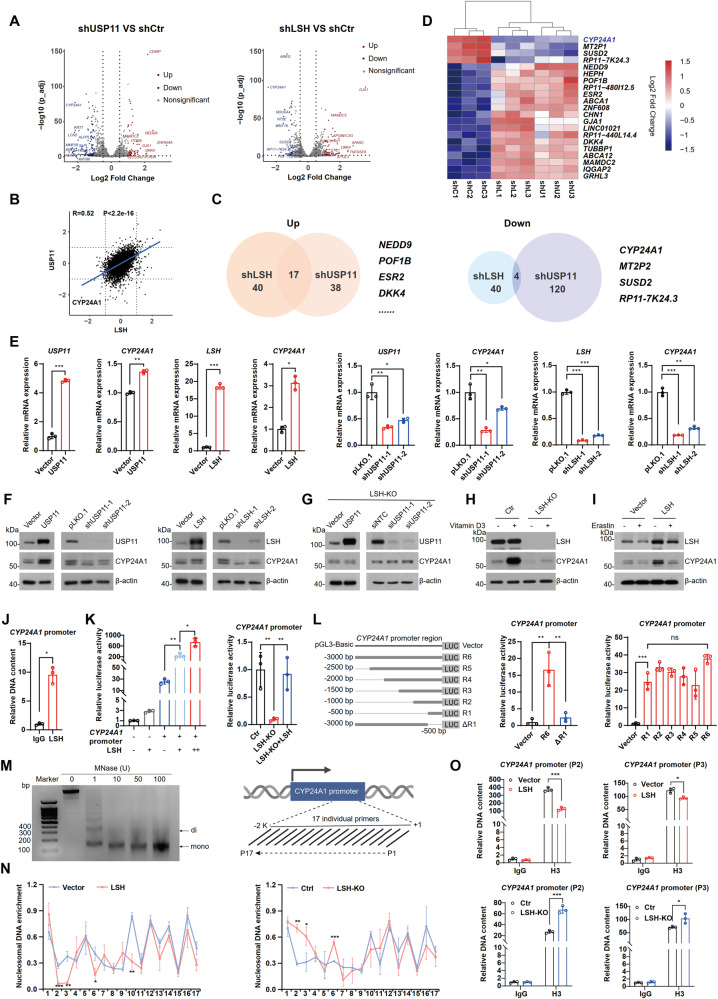
CYP24A1 is a key target of LSH. **A** Volcano plots of DEGs in shUSP11 vs. control (left) and shLSH vs. control (right) HCT116 cells. |Fold change|> 2 and *P* < 0.05 are shown in red (upregulated) or blue (downregulated). **B** Scatter plot showing the fold change in DEGs between shLSH vs. control and shUSP11 vs. control HCT116 cells. The Pearson’s correlation coefficient was calculated. **C** Venn diagrams showing co-upregulated (left) and co-downregulated (right) genes in shUSP11 and shLSH HCT116 cells. **D** Heatmap showing the relative expression of co-upregulated and co-downregulated genes in shUSP11 and shLSH HCT116 cells. **E** RT-qPCR analysis of the expression of CYP24A1 in USP11- or LSH-overexpressing and USP11- or LSH-deficient HCT116 cells. Values are presented as mean ± SD (*n* = 3). **F** WB analysis of the expression of CYP24A1 in USP11- or LSH-overexpressing and USP11- or LSH-deficient HCT116 cells. **G** WB analysis of the expression of CYP24A1 in LSH-KO cells with USP11 overexpression or deficiency. **H** WB and RT-qPCR analysis of the expression of CYP24A1 in control or LSH-KO cells treated with DMSO or vitamin D3 (1 µM) for 24 h. Values are presented as mean ± SD (*n* = 3). **I** WB analysis of the expression of CYP24A1 in control and LSH-overexpressing cells treated with DMSO or erastin (20 µM) for 24 h. **J** ChIP-qPCR showing that LSH binds to the *CYP24A1* promoter in HCT116 cells. Values are presented as mean ± SD (*n* = 3). **K** *CYP24A1* promoter activity was assessed using a dual-luciferase reporter assay in the absence or presence of LSH. Values are presented as mean ± SD (*n* = 3). **L** Schematic diagram of a series of stretches of the sequence upstream of the TSS of *CYP24A1* (left). Luciferase activity assay (right). Values are presented as mean ± SD (*n* = 3). **M** Chromatin from HCT116 cells was incubated with increasing dose of micrococcal nuclease (MNase). Mono-nucleosome sized DNA was visualized by electrophoresis (left). Schematic diagram showing the overlapping primer pairs (typically spaced 30 bp apart) for the 2000 bp promoter region upstream of the TSS of *CYP24A1* (right). **N** Mono-nucleosomes were extracted from gel as template. RT-qPCR analysis of the nucleosome DNA enrichment of *CYP24A1* promoter in LSH-overexpressing and LSH-KO cells. Nucleosome density at a given region was measured as the relative ratio of digested DNA to the undigested control. Values are presented as mean ± SD (*n* = 3). **O** ChIP-qPCR detected the density of H3 histones on *CYP24A1* promoter by P2 and P3 primers as depicted in LSH-overexpressing and LSH-KO cells. Values are presented as mean ± SD (*n* = 3).

To further explore the downstream effectors of the USP11/LSH molecular cascade in ferroptosis, we conducted high-throughput RNA-sequencing analysis in HCT116 cells with manipulated USP11/LSH. Volcano plots showed both upregulated and downregulated differentially expressed genes (DEGs) in USP11- or LSH-deficient cells compared with control cells (Fig. 5A). Changes in transcriptome were similar between USP11 knockdown and LSH knockdown cells (Fig. 5B). Venn diagrams highlighted 17 co-upregulated and 4 co-downregulated DEGs in both USP11-and LSH-deficient cells (Fig. 5C), and the relative expression of these genes was presented in a heatmap (Fig. 5D). The most significantly downregulated gene, *CYP24A1*, was selected and identified. The expression of CYP24A1 increased at both the mRNA and protein levels when USP11 or LSH overexpression and decreased when USP11 or LSH depletion (Figs. 5E, F and S2A, B). However, neither overexpression nor knockdown of USP11 had a substantial impact on CYP24A1 expression in LSH-depleted cells (Fig. 5G), suggesting that *CYP24A1* is a direct transcriptional target of LSH rather than USP11. Previous research has reported that high CYP24A1 expression can be induced by vitamin D3 through the vitamin D3 receptor (VDR) [35]. We confirmed this effect in HCT116 cells, but it was not observed in LSH-depleted cells (Fig. 5H). These results indicate that LSH is required for VDR-mediated *CYP24A1* transcription. Importantly, we found that erastin treatment also degrades CYP24A1, suggesting that CYP24A1 may also participate in erastin-induced ferroptosis (Fig. 5I).

Next, we explored the mechanism by which LSH regulates *CYP24A1* transcription. As shown in Fig. 5J, LSH directly binds to the promoter of the *CYP24A1*. The luciferase reporter assay revealed that the transcriptional activity of *CYP24A1* was significantly enhanced in LSH-overexpressing cells but repressed in LSH-KO cells, which could be rescued by reintroducing LSH (Fig. 5K). Moreover, we identified the 500 bp stretch of DNA upstream of the *CYP24A1* transcriptional start site (TSS) as the primary LSH-binding region (Fig. 5L). Since LSH is a chromatin remodeling factor, we performed MNase mapping to examine the impact of LSH on the chromatin structure of the *CYP24A1* promoter. Chromatin was isolated and 17 individual primer sets were used to amplify the promoter region sequence immediately upstream of the *CYP24A1* TSS (Fig. 5M). As shown in Fig. 5N, enrichment of nucleosomal DNAs at promoter regions P2, P3, and P6 (corresponding to the P1, P2, and P6 primer sets) was significantly decreased in cells overexpressing LSH compared to control, while it was markedly increased in LSH-KO cells. Furthermore, the *CYP24A1* promoter DNAs pulled down by H3 antibody was much less in cells overexpressing LSH but more in LSH-KO cells (Fig. 5O). These findings suggest that LSH enhances *CYP24A1* transcription by promoting nucleosome eviction. In addition, ChIP results indicated altered binding of histones to chromatin DNA during this process. The differential enrichment of H3K27me3 was consistently observed in both LSH-overexpressing cells and LSH-KO cells, suggesting that H3K27me3 might be one of the histone markers primarily affected by LSH (Fig. S2C). Notably, there was no change in the total intracellular H3K27me3 levels in LSH-overexpressing or LSH-depleted cells (Fig. S2D).

### CYP24A1 is required for LSH-mediated inhibition of ferroptosis

**Fig. 6:**
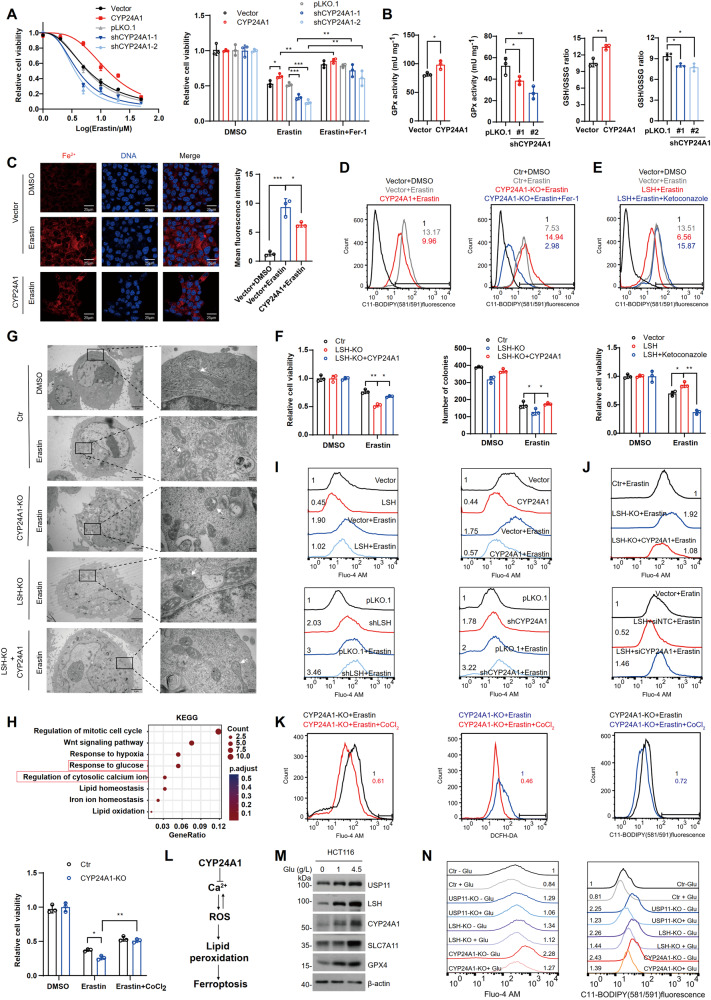
CYP24A1 suppresses ferroptosis by lowering cytosolic Ca^2+^ influx. **A** Cell viability was detected in HCT116 cells overexpressing or deficient in CYP24A1. Cells were treated with different concentrations of erastin for 24 h or erastin (20 μM) for 24 h or Fer-1 (5 μM) for 20 h, Values are presented as mean ± SD (*n* = 3). **B** Intracellular GPx activity and GSH and GSSG levels were assessed in HCT116 cells overexpressing or deficient in CYP24A1. Values are presented as mean ± SD (*n* = 3). **C** Intracellular Fe^2+^ levels were examined in control and CYP24A1-overexpressing HCT116 cells treated with DMSO or erastin (20 μM) for 24 h. Scale bar, 25 µm. **D** Lipid peroxidation was assessed in HCT116 cells overexpressing or deficient in CYP24A1 treated with erastin (20 μM) for 24 h or Fer-1 (5 μM) for 20 h. **E** Lipid peroxidation (top) and cell viability (bottom) were assessed in the indicated cells treated with erastin (20 µM) for 24 h or ketoconazole (5 µM) for 8 h. Values are presented as mean ± SD (*n* = 3). **F** Cell viability (left) and colony formation capacity (right) were examined in the indicated cells treated with DMSO or erastin (20 µM) for 24 h. Values are presented as mean ± SD (*n* = 3). **G** Transmission electron microscopy analysis of mitochondria in indicated cells. The white arrow indicates the mitochondria. Scale bar, 2 µm (left) and 200 nm (right). **H** GO analysis of co-downregulated genes from shUSP11 and shLSH cells enriched in physiological signaling pathways. The *P*-value was calculated using a one-tailed Fisher’s exact test. **I**, **J** Intracellular Ca^2+^ levels were determined in the indicated cells treated with DMSO or erastin (20 µM) for 24 h. **K** Intracellular Ca^2+^, ROS, lipid peroxidation levels, and cell viability were assessed in CYP24A1-KO cells treated with erastin (20 µM) for 24 h or CoCl_2_ (50 μM) for 24 h. Values are presented as mean ± SD (*n* = 3). **L** Schematic model of CYP24A1-mediated ferroptosis inhibition. **M** HCT116 cells were incubated with different concentrations of glucose and lysed for WB analysis. **N** Intracellular Ca^2+^ levels (left) and lipid peroxidation (right) were assessed in cells cultured in normal (+Glu) or glucose-free (−Glu) media and treated with erastin (20 µM) for 24 h.

Next, we investigated whether CYP24A1 plays a role in ferroptosis. Cell viability analysis revealed that CYP24A1 overexpression conferred cells with enhanced resistance to erastin-induced ferroptosis, whereas its deficiency re-sensitized the cells to ferroptosis, which could be restored by Fer-1 treatment (Fig. 6A). Moreover, overexpression of CYP24A1 prevented ferroptotic events, including elevated GPx enzymatic activity and GSH levels, and reduced intracellular GSSG, Fe^2+^, and lipid peroxidation levels following erastin treatment (Fig. 6B–D). Conversely, CYP24A1 deficiency had the opposite effects (Fig. 6B, D). Notably, inhibiting CYP24A1 activity with ketoconazole nullified the effect of LSH on reducing lipid peroxidation and increasing cell viability after erastin treatment (Fig. 6E). In contrast, CYP24A1 overexpression partially restored viability and colony formation ability in LSH-KO cells (Fig. 6F). Moreover, transmission electron microscopy (TEM) was performed to examine morphological and structural changes in mitochondria during ferroptosis. The results showed that HCT116 cells exhibited shrunken mitochondria with increased membrane density and decreased cristae upon erastin treatment, which are classical features of ferroptosis; whereas control cells did not display such changes (Fig. 6G). Importantly, LSH-KO and CYP24A1-KO cells (Fig. S1C) exhibited exacerbated ferroptosis characteristics, and these changes were largely diminished by re-expressing CYP24A1 in LSH-KO cells (Fig. 6G). Overall, these findings imply that CYP24A1 is an indispensable downstream effector of LSH in suppressing ferroptosis.

### CYP24A1 inhibits ferroptosis by lowering cytosolic Ca^2+^ influx

CYP24A1 is an enzyme responsible for deactivating vitamin D precursor (25(OH)D) and active vitamin D (1α,25(OH)_2_D), and its dysregulation leads to vitamin D and calcium metabolism disorders [36]. It is worth noting that calcium abnormalities can activate cell death signaling [37]. Hence, we hypothesized that CYP24A1 might influence ferroptosis by modulating cellular calcium levels. Gene ontology (GO) analysis of our transcriptomic dataset revealed enrichment of the calcium regulation pathway (Fig. 6H). Indeed, erastin-induced abnormal cytoplasmic Ca^2+^ fluxes, and overexpression of LSH or CYP24A1 reduced Ca^2+^ levels to varying extents during ferroptosis in HCT116 cells, whereas their deficiency produced opposite effects (Fig. 6I). Moreover, CYP24A1 overexpression partially suppressed erastin-induced Ca^2+^ elevation in LSH-depleted cells, whereas CYP24A1 knockdown in LSH-overexpressing cells resulted in elevated Ca^2+^ levels (Fig. 6J). However, blocking Ca^2+^ with CoCl_2_ [38] markedly alleviated erastin sensitivity, leading to reduced ROS, decreased lipid peroxidation, and restored viability in CYP24A1-KO cells (Fig. 6K, L). These results suggest that CYP24A1 mitigates the progressive increase in ROS and oxidative stress by lowering cytosolic Ca^2+^ during erastin-induced ferroptosis.

Interestingly, GO analysis also indicated a potential association between glucose metabolism and the USP11/LSH/CYP24A1 axis. The protein expression of USP11, LSH, and CYP24A1 were significantly decreased in response to glucose starvation (Fig. 6M). Importantly, higher levels of Ca^2+^ and lipid peroxidation were observed when glucose depletion reduced USP11/LSH/CYP24A1 protein abundance (Fig. 6N), indicating that the ability of USP11/LSH/CYP24A1 to inhibit ferroptosis may be sensitive to glucose deficiency.

### The USP11/LSH/CYP24A1 axis is aberrantly activated in CRC

**Fig. 7:**
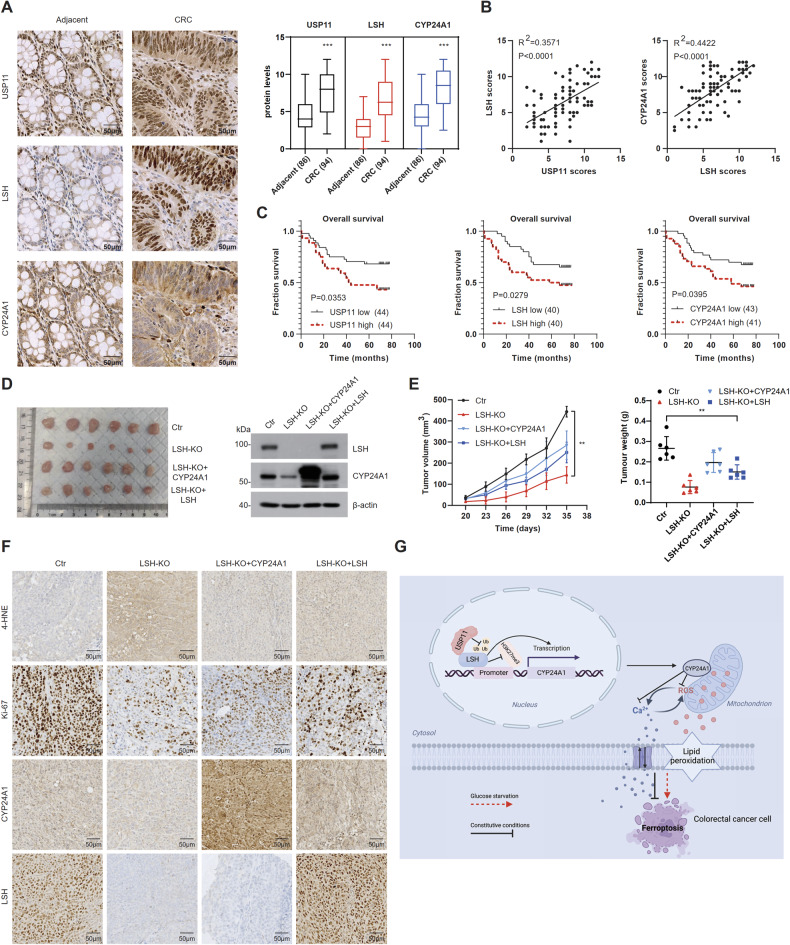
The USP11/LSH/CYP24A1 axis is aberrantly activated in CRC. **A** Immunohistochemical analysis of USP11, LSH, and CYP24A1 expression in human CRC and adjacent normal tissues. Scale bar, 50 μm. **B** Correlations between USP11 and LSH and between LSH and CYP24A1 were analyzed by Linear regression analysis (*n* = 94). **C** Analysis of CRC patient prognosis stratified by USP11, LSH, and CYP24A1 expression levels. **D** Tumor formation in the xenografted mice (*n* = 6 per group) (left). WB analysis of the indicated proteins (right). **E** Tumor growth rate of xenografts in BALB/c nude mice. Tumor weight and volumes were measured and plotted. Values are presented as mean ± SD (*n* = 6). **F** Immunohistochemical analysis of tumor xenografts. Scale bar, 50 μm. **G** Schematic model of USP11/LSH/CYP24A1 axis-mediated resistance to ferroptosis in CRC cells.

To elucidate the oncogenic potential of the USP11/LSH/CYP24A1 axis in CRC, we conducted immunohistochemical staining on tissue microarrays (TMAs). The results revealed high expression of these three proteins in CRC tissues (Fig. 7A), and positive correlations were observed between USP11 and LSH, as well as between LSH and CYP24A1 (Fig. 7B). Furthermore, increased expression of USP11, LSH, and CYP24A1 was associated with shorter survival in CRC patients (Fig. 7C). In animal studies, we found that LSH-KO cell-derived xenografts showed slower growth rates, much smaller tumor volumes than control cells, and displayed increased 4-HNE (4-hydroxy-2-noneal, a well-known marker for lipid peroxides) [39] and decreased Ki-67 staining. However, restoration of CYP24A1 or LSH partially reversed these phenotypes (Fig. 7D–F). Taken together, the aberrant activation of the USP11/LSH/CYP24A1 pathway may play a critical role in the malignant progression of CRC through regulating ferroptosis.

## Discussion

LSH is a chromatin-remodeling factor that regulates the epigenetic modification of chromatin through its association with various factors, such as chromatin modification enzymes, transcription factors, transcription cofactors, and repressors [16]. Recent studies have highlighted the role of LSH in suppressing ferroptosis in leukemia and lung cancer [21, 23, 24]. Consistent with these findings, our study demonstrates a similar role for LSH in inhibiting ferroptosis in CRC. One of the mechanisms by which LSH regulates ferroptosis is through the transcriptional regulation of genes involved in cellular lipid and iron metabolism, which ultimately impacts lipid peroxidation and iron levels. To date, several metabolism-related genes, including *SLC7A11, ACSL4, ALOX5AP, SCD1, FADS2*, and *FTH1* [21, 22, 33], have been identified as targets of LSH. Apart from these genes, we identified *CYP24A1* as a novel target gene for LSH and demonstrated its critical role in LSH-regulated ferroptosis.

CYP24A1 primarily localizes to the inner mitochondrial membrane and is involved in regulating calcium metabolism [40, 41]. Mutations or deletions in *CYP24A1* are associated with hypercalcemia and hypercalciuria [42, 43]. However, the role of CYP24A1 in regulating ferroptosis has not yet been reported. Here, we demonstrated that CYP24A1 inhibits ferroptosis by preventing intracellular calcium overload. Mechanistically, CYP24A1 reduces cytoplasmic Ca^2+^ during ferroptosis, thereby suppressing the increase in ROS and lipid peroxidation, and ultimately reducing the sensitivity to ferroptosis. Previous studies have also shown that dysregulation of calcium metabolism is closely associated with ferroptosis. Elevated cytoplasmic Ca^2+^ has been shown to promote the insertion of 12/15-lipoxygenase into the membranes of the endoplasmic reticulum and mitochondria, which causes membrane lipid peroxidation and leads to ferroptosis [44, 45]. In contrast, cobalt chloride, apomorphine, and LY83583, which reduce Ca^2+^ influx protect cells from erastin-induced ferroptosis [46]. Emerging evidence suggests that an imbalance in Ca^2+^ influx may be a promoter of ferroptosis. For instance, a persistent elevation in cytosolic Ca^2+^ levels in NIH-3T3 cells indicates complete cell rupture resulting from ferroptosis [47]. Moreover, MICU1-dependent mitochondrial Ca^2+^ uptake and mitochondrial membrane potential hyperpolarization promote ferroptosis upon cold stimulation [48]. Besides, MS4A15 specifically blocks ferroptosis by balancing ER Ca^2+^-induced lipid remodeling [49]. These findings support a role for cytosolic Ca^2+^ elevation in the promotion of ferroptosis.

In addition to its role in regulating cellular calcium levels, CYP24A1 may regulate lipid peroxidation by maintaining redox homeostasis and ultimately affecting ferroptosis. CYP24A1 is a cytochrome P450 monooxygenase that belongs to the cytochrome P450 oxidoreductase (POR) family [50]. POR generates hydrogen peroxide by transferring electrons from NAD(P)H to oxygen, which subsequently enhances the generation of reactive hydroxyl radicals and promotes peroxidation of polyunsaturated fatty acids in phospholipids [51, 52].

Given the central role of LSH in regulating ferroptosis, factors that influence LSH protein homeostasis may also impact ferroptosis. Previous studies have shown that the long noncoding RNA LINC00618 suppresses LSH expression and promotes ferroptosis [21], while EGLN1 and c-Myc, which activate *LSH* transcription, inhibit ferroptosis [22]. In our study, we identified USP11 as a crucial DUB that stabilizes LSH through deubiquitination. USP11 mitigates erastin-induced LSH degradation, thereby strengthening the resistance of CRC cells to ferroptosis. Consistent with our findings, USP11 has recently been shown to inhibit ferroptosis by stabilizing NRF2 protein levels and promoting the proliferation of lung carcinoma cells under oxidative stress [53]. Conversely, E3 ubiquitin ligases involved in LSH degradation may also play a role in ferroptosis. A recent study reported that the E3 ligase CRL4^DCAF8^ participates in the proteolysis of LSH during ferroptosis [33]. However, how erastin induces DCAF8-dependent LSH protein degradation remains unknown. Ubiquitination and deubiquitination processes collaborate to maintain protein homeostasis, and further investigations are needed to clarify the role of E3 ubiquitin ligases in LSH degradation during erastin-induced ferroptosis.

Dysregulation of the USP11/LSH/CYP24A1 pathway may be a universal phenomenon in multiple cancers. USP11 overexpression is widespread in tumors and has been identified as a diagnostic or prognostic marker for certain cancers [54]. LSH shows significant upregulation in various cancers and is associated with poor prognosis and short survival [16]. Similarly, CYP24A1 is aberrantly expressed at high levels and has been implicated as a potential oncogene in colorectal, breast, and other cancers [55]. These findings highlight the close association between dysregulation of the USP11/LSH/CYP24A1 pathway and tumorigenesis and have implications for cancer treatment and prevention.

In summary, we propose a novel regulatory pathway that elucidates the role of LSH in suppressing ferroptosis in CRC (Fig. 7G). LSH is stabilized by USP11 and promotes CYP24A1 transcription through a mechanism dependent of epigenetic regulation. The USP11/LSH/CYP24A1 cascade helps CRC cells resist ferroptosis by modulating abnormal Ca^2+^ fluxes. However, glucose starvation promotes ferroptosis by decreasing these three proteins’ abundance in CRC cells. Therefore, future studies on manipulating the glucose microenvironment, combined with targeting the USP11/LSH/CYP24A1 signaling axis to enhance cell sensitivity to ferroptosis, may have important implications for the treatment of CRC.

## Supplementary information

Supplementary Figure S1

Supplementary Figure S2

Supplementary table information

Original western blots

aj-checklist

## Data Availability

RNA-sequencing data in this study are available in the Gene Expression Omnibus (GEO) database (http://www.ncbi.nlm.nih.gov/geo/) under accession number GSE211866. The data used in this study are available in the manuscript and the supplementary files.

## References

[1] Chen X, Li J, Kang R, Klionsky DJ, Tang D (2021). Ferroptosis: machinery and regulation. Autophagy.

[2] Tang D, Chen X, Kang R, Kroemer G (2021). Ferroptosis: molecular mechanisms and health implications. Cell Res.

[3] Jiang X, Stockwell BR, Conrad M (2021). Ferroptosis: mechanisms, biology and role in disease. Nat Rev Mol Cell Biol.

[4] Doll S, Freitas FP, Shah R, Aldrovandi M, da Silva MC, Ingold I (2019). FSP1 is a glutathione-independent ferroptosis suppressor. Nature.

[5] Mao C, Liu X, Zhang Y, Lei G, Yan Y, Lee H (2021). DHODH-mediated ferroptosis defence is a targetable vulnerability in cancer. Nature.

[6] Kraft VAN, Bezjian CT, Pfeiffer S, Ringelstetter L, Müller C, Zandkarimi F (2020). GTP cyclohydrolase 1/tetrahydrobiopterin counteract ferroptosis through lipid remodeling. ACS Cent Sci.

[7] Stockwell BR, Friedmann Angeli JP, Bayir H, Bush AI, Conrad M, Dixon SJ (2017). Ferroptosis: a regulated cell death nexus linking metabolism, redox biology, and disease. Cell.

[8] Stockwell BR (2022). Ferroptosis turns 10: emerging mechanisms, physiological functions, and therapeutic applications. Cell.

[9] Chen X, Kang R, Kroemer G, Tang D (2021). Broadening horizons: the role of ferroptosis in cancer. Nat Rev Clin Oncol.

[10] Liang C, Zhang X, Yang M, Dong X (2019). Recent progress in ferroptosis inducers for cancer therapy. Adv Mater.

[11] Wang W, Green M, Choi JE, Gijón M, Kennedy PD, Johnson JK (2019). CD8(+) T cells regulate tumour ferroptosis during cancer immunotherapy. Nature.

[12] Lang X, Green MD, Wang W, Yu J, Choi JE, Jiang L (2019). Radiotherapy and immunotherapy promote tumoral lipid oxidation and ferroptosis via synergistic repression of SLC7A11. Cancer Discov.

[13] Schwartz AJ, Goyert JW, Solanki S, Kerk SA, Chen B, Castillo C (2021). Hepcidin sequesters iron to sustain nucleotide metabolism and mitochondrial function in colorectal cancer epithelial cells. Nat Metab.

[14] Basak D, Uddin MN, Hancock J (2020). The role of oxidative stress and its counteractive utility in colorectal cancer (CRC). Cancers.

[15] Dürr H, Flaus A, Owen-Hughes T, Hopfner KP (2006). Snf2 family ATPases and DExx box helicases: differences and unifying concepts from high-resolution crystal structures. Nucleic Acids Res.

[16] Chen X, Li Y, Rubio K, Deng B, Li Y, Tang Q (2022). Lymphoid-specific helicase in epigenetics, DNA repair and cancer. Br J Cancer.

[17] Xu X, Ni K, He Y, Ren J, Sun C, Liu Y (2021). The epigenetic regulator LSH maintains fork protection and genomic stability via MacroH2A deposition and RAD51 filament formation. Nat Commun.

[18] Geiman TM, Muegge K (2000). Lsh, an SNF2/helicase family member, is required for proliferation of mature T lymphocytes. Proc Natl Acad Sci USA.

[19] He Y, Ren J, Xu X, Ni K, Schwader A, Finney R (2020). Lsh/HELLS is required for B lymphocyte development and immunoglobulin class switch recombination. Proc Natl Acad Sci USA.

[20] Thijssen PE, Ito Y, Grillo G, Wang J, Velasco G, Nitta H (2015). Mutations in CDCA7 and HELLS cause immunodeficiency-centromeric instability-facial anomalies syndrome. Nat Commun.

[21] Wang Z, Chen X, Liu N, Shi Y, Liu Y, Ouyang L (2021). A nuclear long non-coding RNA LINC00618 accelerates ferroptosis in a manner dependent upon apoptosis. Mol Ther.

[22] Jiang Y, Mao C, Yang R, Yan B, Shi Y, Liu X (2017). EGLN1/c-Myc induced lymphoid-specific helicase inhibits ferroptosis through lipid metabolic gene expression changes. Theranostics.

[23] Wang M, Mao C, Ouyang L, Liu Y, Lai W, Liu N (2019). Long noncoding RNA LINC00336 inhibits ferroptosis in lung cancer by functioning as a competing endogenous RNA. Cell Death Differ.

[24] Mao C, Wang X, Liu Y, Wang M, Yan B, Jiang Y (2018). A G3BP1-interacting lncRNA promotes ferroptosis and apoptosis in cancer via nuclear sequestration of p53. Cancer Res.

[25] Petesch SJ, Lis JT (2008). Rapid, transcription-independent loss of nucleosomes over a large chromatin domain at Hsp70 loci. Cell.

[26] Li Q, Zhang Y, Fu J, Han L, Xue L, Lv C (2013). FOXA1 mediates p16(INK4a) activation during cellular senescence. EMBO J.

[27] Zhang Y, Shi J, Liu X, Feng L, Gong Z, Koppula P (2018). BAP1 links metabolic regulation of ferroptosis to tumour suppression. Nat Cell Biol.

[28] Yang WS, SriRamaratnam R, Welsch ME, Shimada K, Skouta R, Viswanathan VS (2014). Regulation of ferroptotic cancer cell death by GPX4. Cell.

[29] Yang WS, Stockwell BR (2016). Ferroptosis: death by lipid peroxidation. Trends Cell Biol.

[30] Shen Z, Song J, Yung BC, Zhou Z, Wu A, Chen X (2018). Emerging strategies of cancer therapy based on ferroptosis. Adv Mater.

[31] Hassannia B, Vandenabeele P, Vanden Berghe T (2019). Targeting ferroptosis to iron out cancer. Cancer Cell.

[32] Harper S, Gratton HE, Cornaciu I, Oberer M, Scott DJ, Emsley J (2014). Structure and catalytic regulatory function of ubiquitin specific protease 11 N-terminal and ubiquitin-like domains. Biochemistry.

[33] Huang D, Li Q, Sun X, Sun X, Tang Y, Qu Y (2021). CRL4(DCAF8) dependent opposing stability control over the chromatin remodeler LSH orchestrates epigenetic dynamics in ferroptosis. Cell Death Differ.

[34] Burkhart RA, Peng Y, Norris ZA, Tholey RM, Talbott VA, Liang Q (2013). Mitoxantrone targets human ubiquitin-specific peptidase 11 (USP11) and is a potent inhibitor of pancreatic cancer cell survival. Mol Cancer Res.

[35] Luo W, Johnson CS, Trump DL (2016). Vitamin D signaling modulators in cancer therapy. Vitamins Hormones.

[36] Christakos S, Dhawan P, Verstuyf A, Verlinden L, Carmeliet G (2016). Vitamin D: metabolism, molecular mechanism of action, and pleiotropic effects. Physiol Rev.

[37] Danese A, Leo S, Rimessi A, Wieckowski MR, Fiorica F, Giorgi C (2021). Cell death as a result of calcium signaling modulation: a cancer-centric prospective. Biochim Biophys Acta Mol Cell Res.

[38] Gleitze S, Paula-Lima A, Núñez MT, Hidalgo C (2021). The calcium-iron connection in ferroptosis-mediated neuronal death. Free Radic Biol Med.

[39] Zhong H, Yin H (2015). Role of lipid peroxidation derived 4-hydroxynonenal (4-HNE) in cancer: focusing on mitochondria. Redox Biol.

[40] Annalora AJ, Goodin DB, Hong WX, Zhang Q, Johnson EF, Stout CD (2010). Crystal structure of CYP24A1, a mitochondrial cytochrome P450 involved in vitamin D metabolism. J Mol Biol.

[41] Garbincius JF, Elrod JW (2022). Mitochondrial calcium exchange in physiology and disease. Physiol Rev.

[42] Schlingmann KP, Kaufmann M, Weber S, Irwin A, Goos C, John U (2011). Mutations in CYP24A1 and idiopathic infantile hypercalcemia. New Engl J Med.

[43] Sy-Go JPT, Zand L, Harris PC, Lieske JC (2021). CYP24A1 deficiency causing persistent hypercalciuria in a stone former. J Nephrol.

[44] Brinckmann R, Schnurr K, Heydeck D, Rosenbach T, Kolde G, Kühn H (1998). Membrane translocation of 15-lipoxygenase in hematopoietic cells is calcium-dependent and activates the oxygenase activity of the enzyme. Blood.

[45] van Leyen K, Duvoisin RM, Engelhardt H, Wiedmann M (1998). A function for lipoxygenase in programmed organelle degradation. Nature.

[46] Maher P, van Leyen K, Dey PN, Honrath B, Dolga A, Methner A (2018). The role of Ca(2+) in cell death caused by oxidative glutamate toxicity and ferroptosis. Cell Calcium.

[47] Pedrera L, Espiritu RA, Ros U, Weber J, Schmitt A, Stroh J (2021). Ferroptotic pores induce Ca(2+) fluxes and ESCRT-III activation to modulate cell death kinetics. Cell Death Differ.

[48] Nakamura T, Ogawa M, Kojima K, Takayanagi S, Ishihara S, Hattori K (2021). The mitochondrial Ca(2+) uptake regulator, MICU1, is involved in cold stress-induced ferroptosis. EMBO Rep.

[49] Xin S, Mueller C, Pfeiffer S, Kraft VAN, Merl-Pham J, Bao X (2022). MS4A15 drives ferroptosis resistance through calcium-restricted lipid remodeling. Cell Death Differ.

[50] Jones G, Prosser DE, Kaufmann M (2012). 25-Hydroxyvitamin D-24-hydroxylase (CYP24A1): its important role in the degradation of vitamin D. Arch Biochem Biophys.

[51] Zou Y, Li H, Graham ET, Deik AA, Eaton JK, Wang W (2020). Cytochrome P450 oxidoreductase contributes to phospholipid peroxidation in ferroptosis. Nat Chem Biol.

[52] Yan B, Ai Y, Sun Q, Ma Y, Cao Y, Wang J (2021). Membrane damage during ferroptosis is caused by oxidation of phospholipids catalyzed by the oxidoreductases POR and CYB5R1. Mol Cell.

[53] Meng C, Zhan J, Chen D, Shao G, Zhang H, Gu W (2021). The deubiquitinase USP11 regulates cell proliferation and ferroptotic cell death via stabilization of NRF2 USP11 deubiquitinates and stabilizes NRF2. Oncogene.

[54] Guo T, Tang H, Yuan Z, Zhang E, Wang X (2022). The Dual Role of USP11 in Cancer. J Oncol.

[55] Sakaki T, Yasuda K, Kittaka A, Yamamoto K, Chen TC (2014). CYP24A1 as a potential target for cancer therapy. Anticancer Agents Med Chem.

