# Supplementary figures and images for: USP11-mediated LSH deubiquitination inhibits ferroptosis in colorectal cancer through epigenetic activation of CYP24A1
Source: Cell Death Dis. 2023 Jul 6;14(7):402. doi: 10.1038/s41419-023-05915-9 (PMC10326026; doi:10.1038/s41419-023-05915-9)

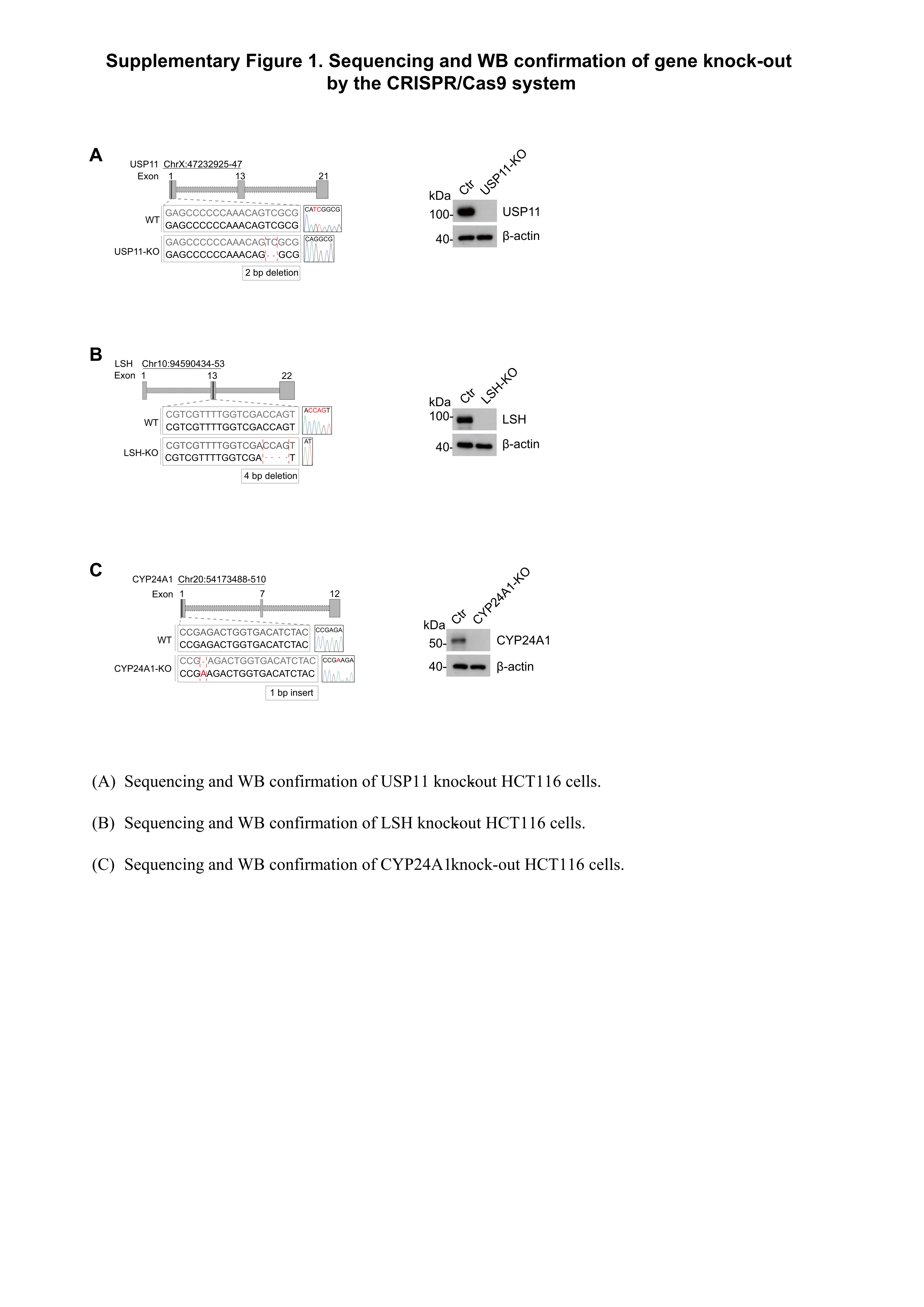

Supplement: Supplementary file 1 — Supplementary Figure S1 [file 41419_2023_5915_MOESM1_ESM.tif]

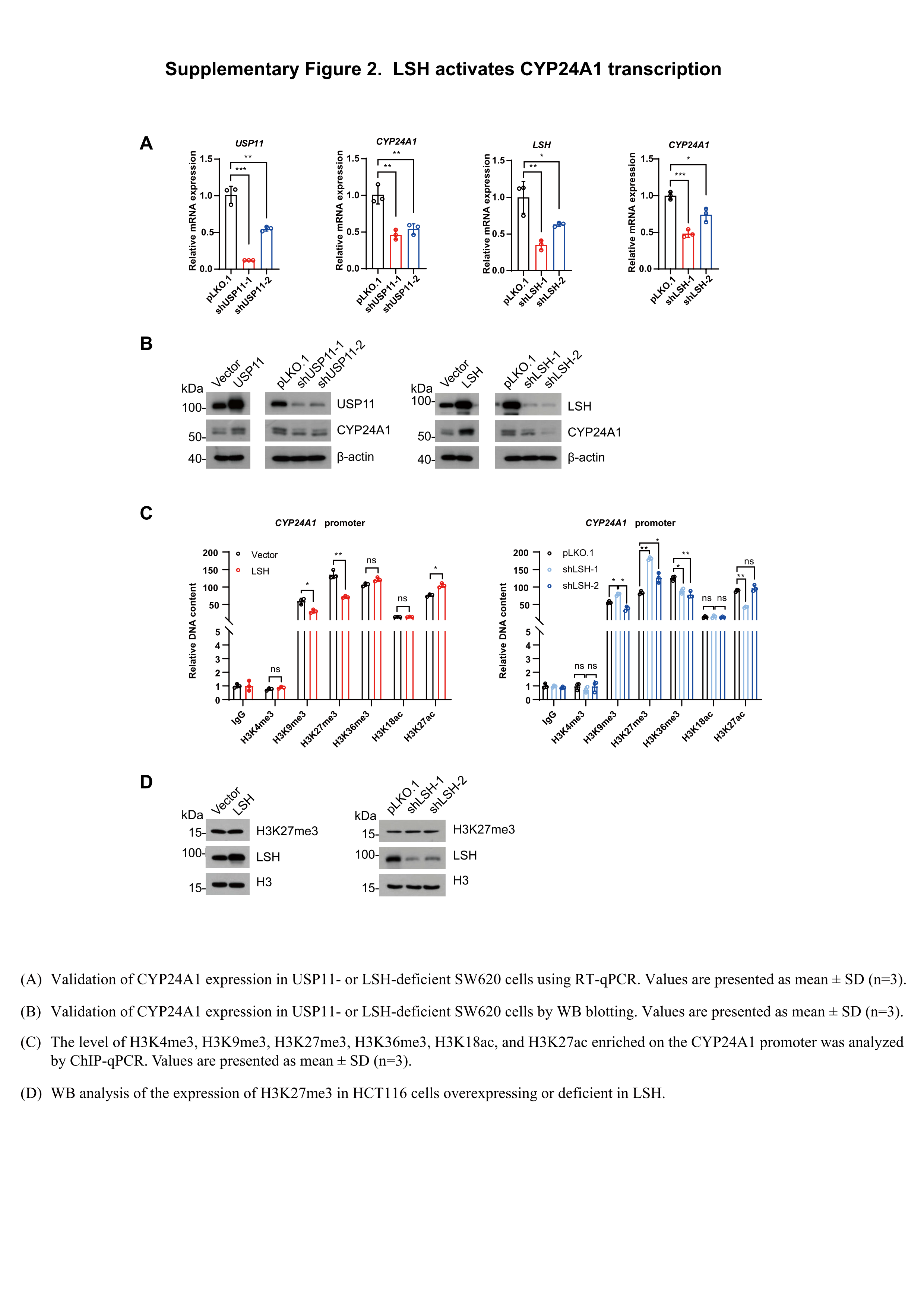

Supplement: Supplementary file 2 — Supplementary Figure S2 [file 41419_2023_5915_MOESM2_ESM.tif]

**1B**

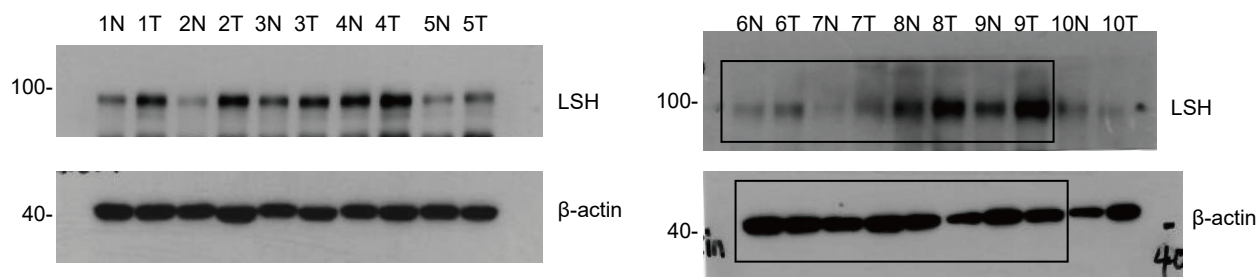

**1E**

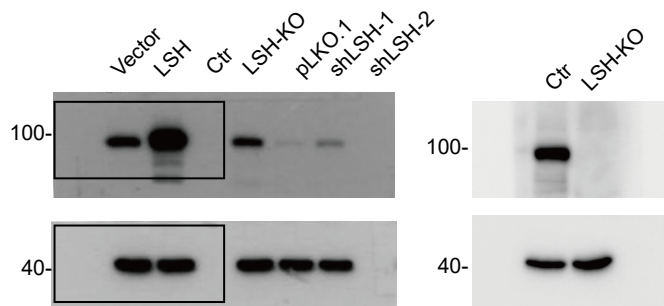

**1I**

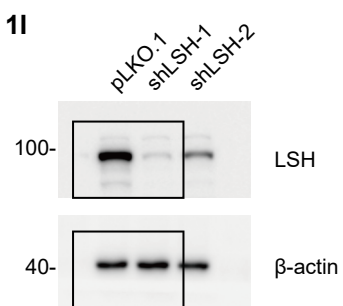

2A

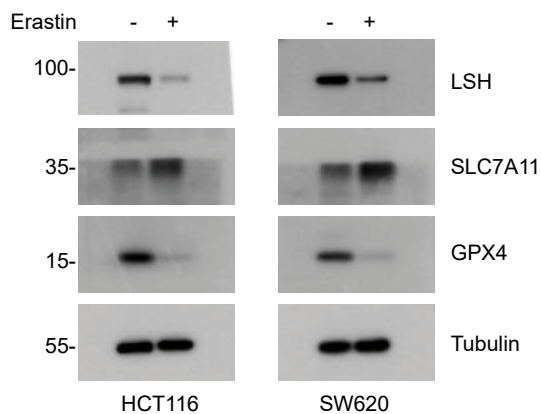

2B

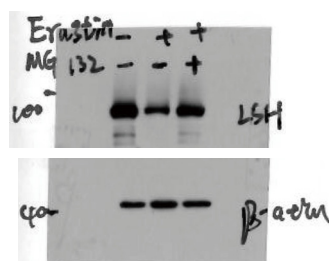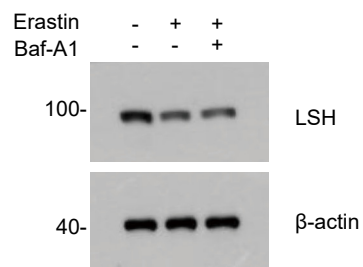

2C

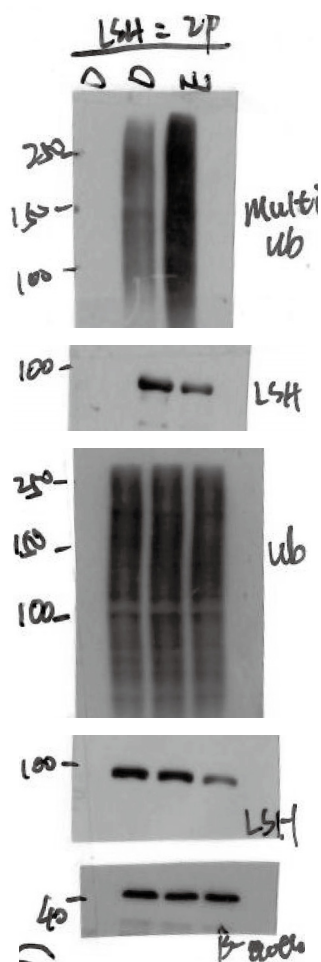

2D

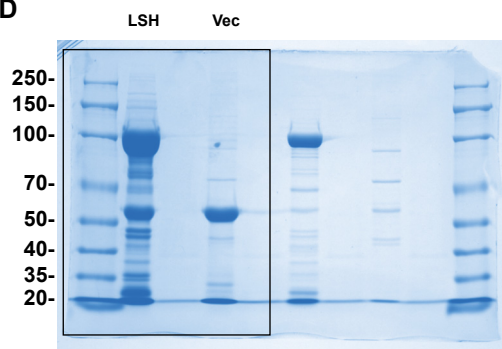

2E

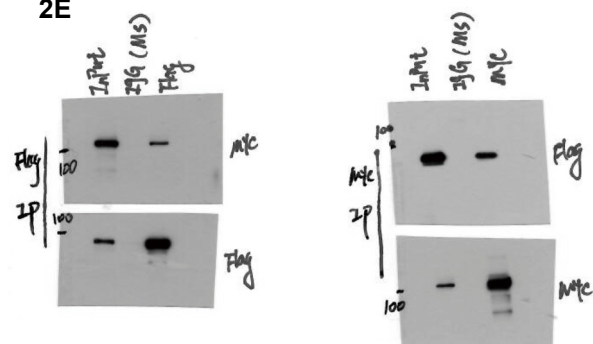

2F

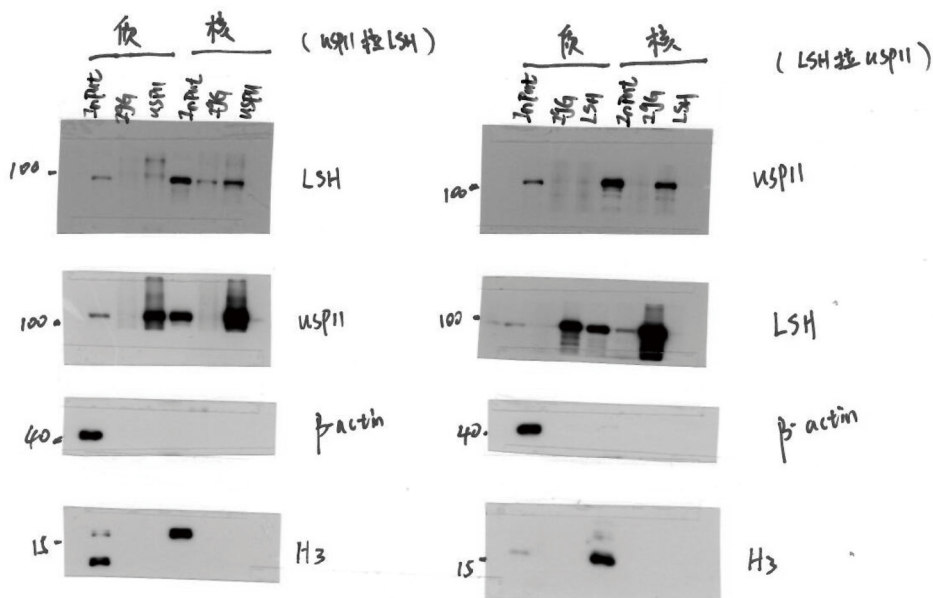

2H

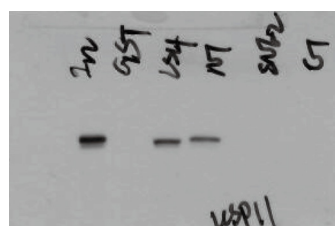

GST FL NT SNF2 CT

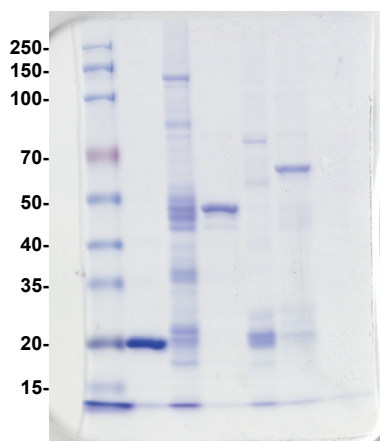

3A

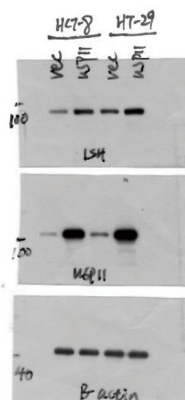

3B

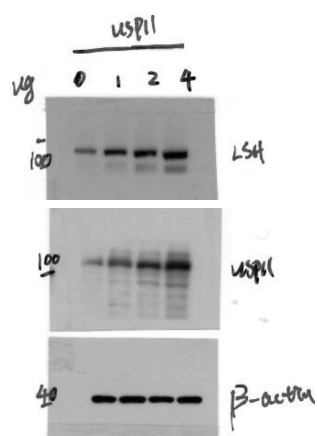

3C

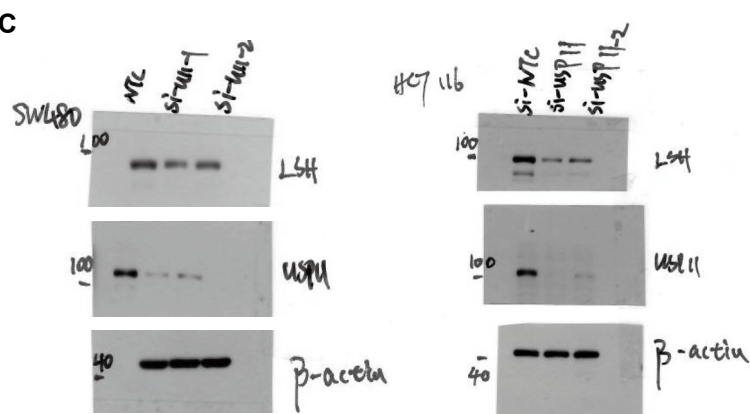

3D &amp; 3E

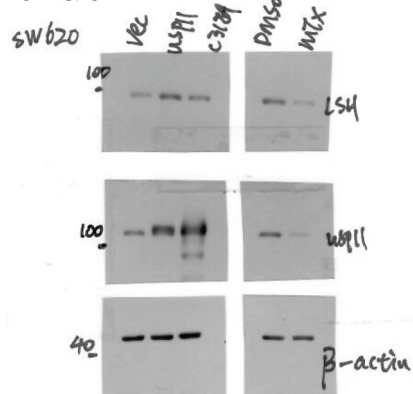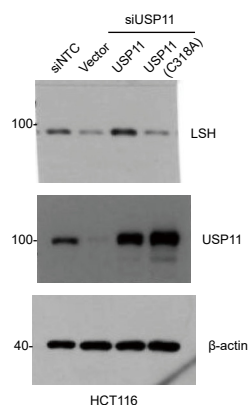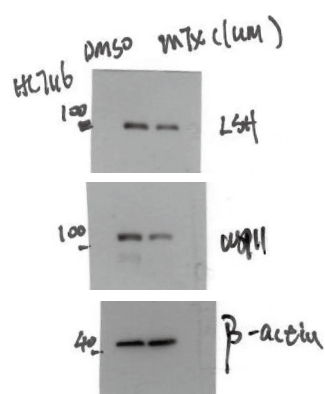

3F

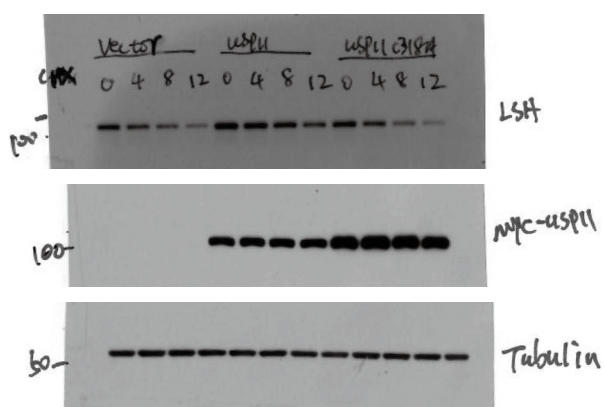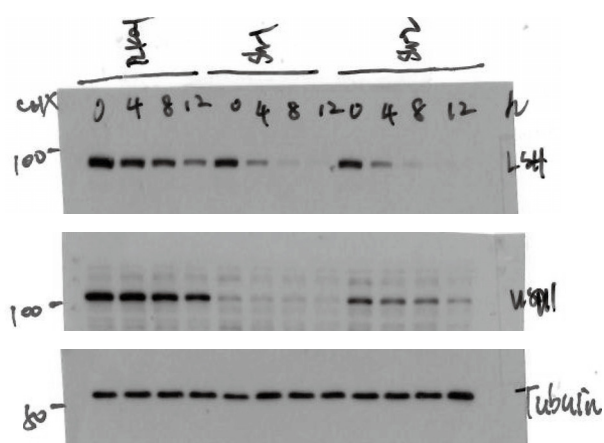

3G

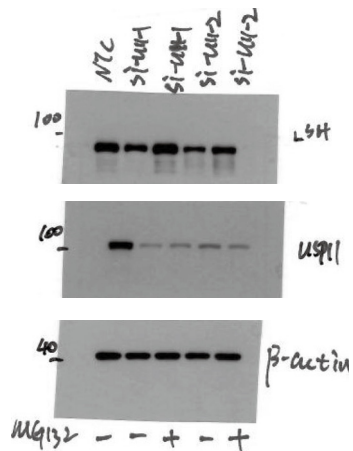

3H

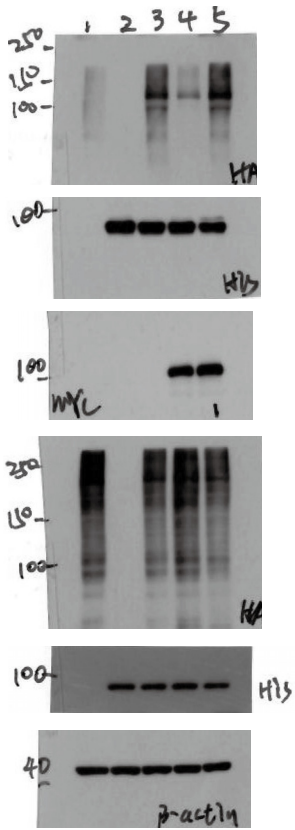

3I

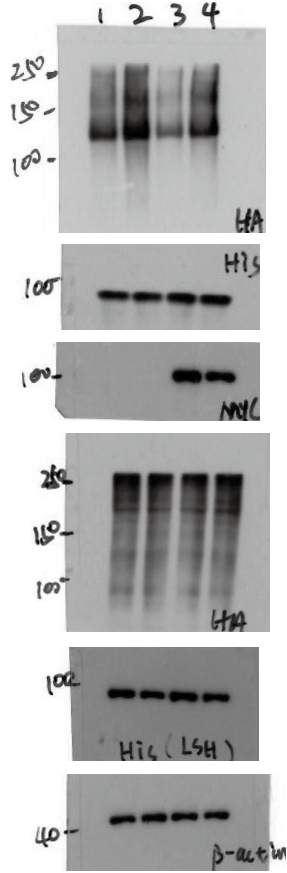

3J

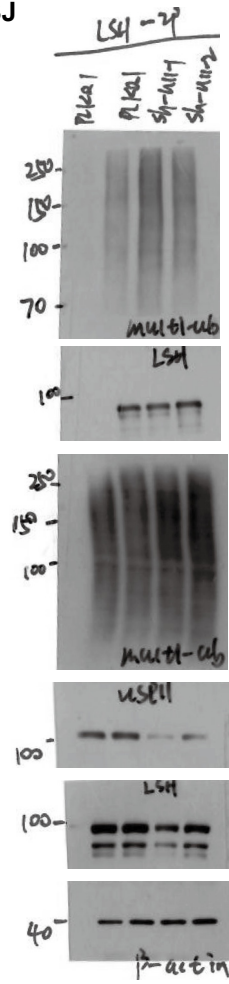

3K

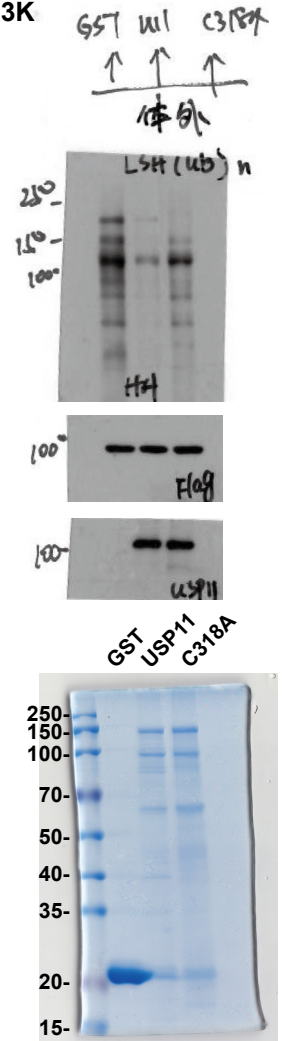

3L

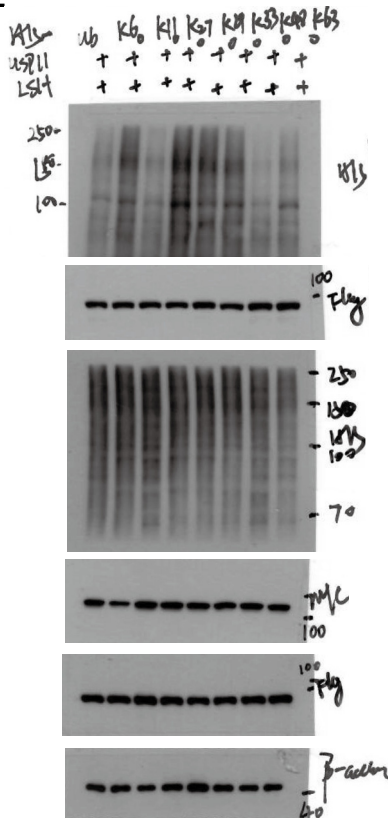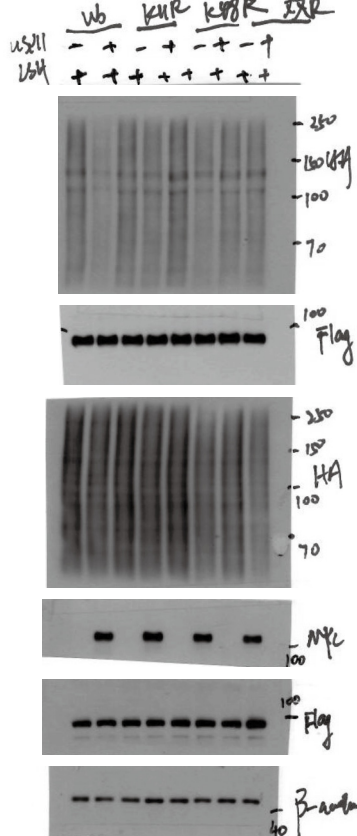

3M

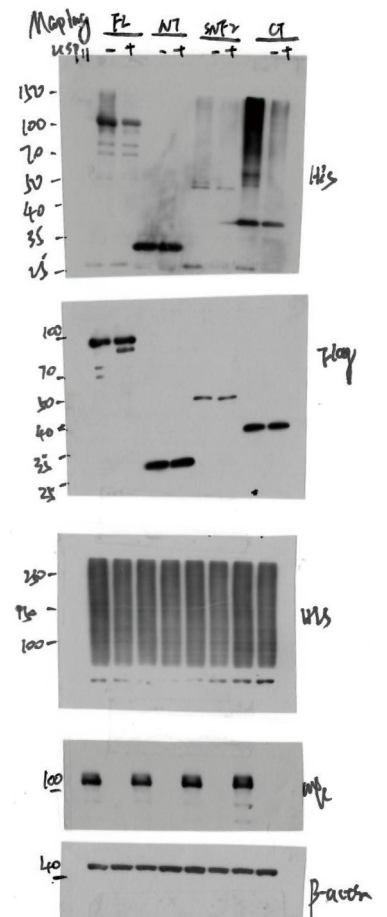

**4A**

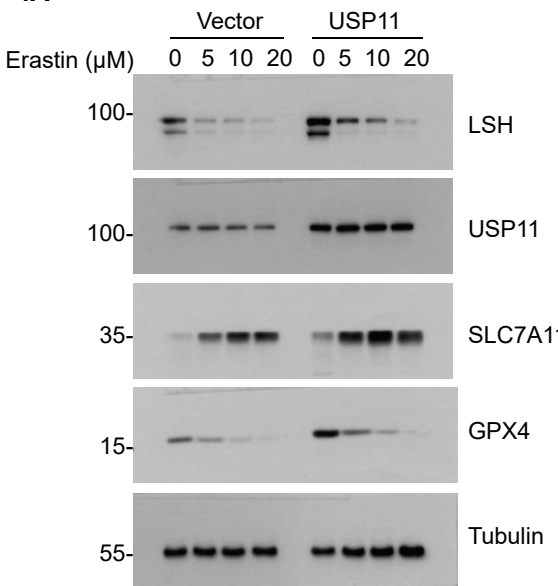

**4B**

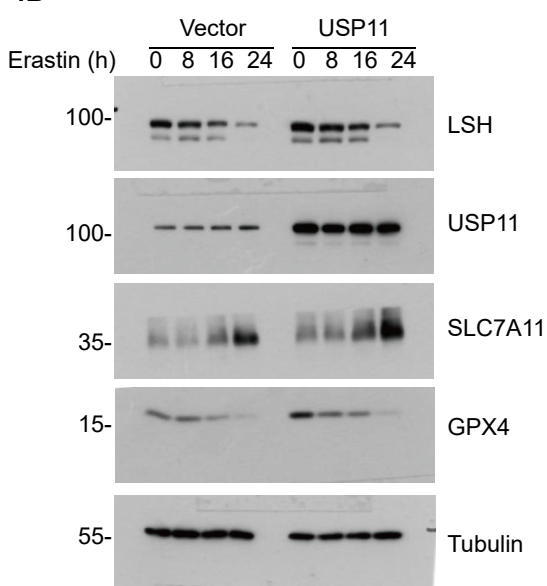

**4F**

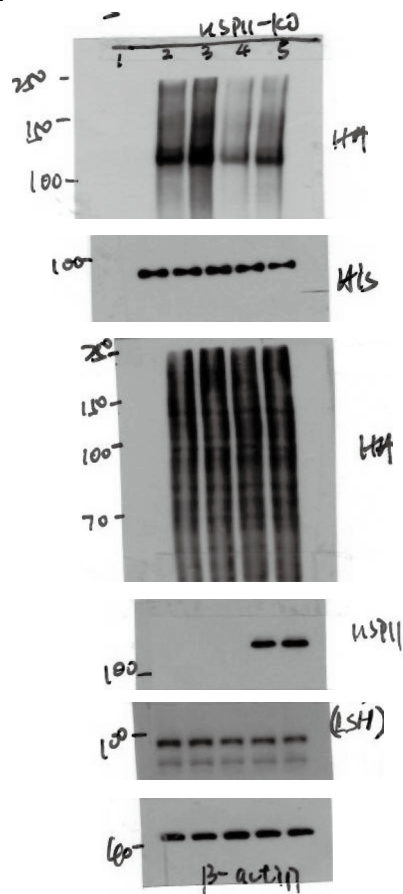

## 4C

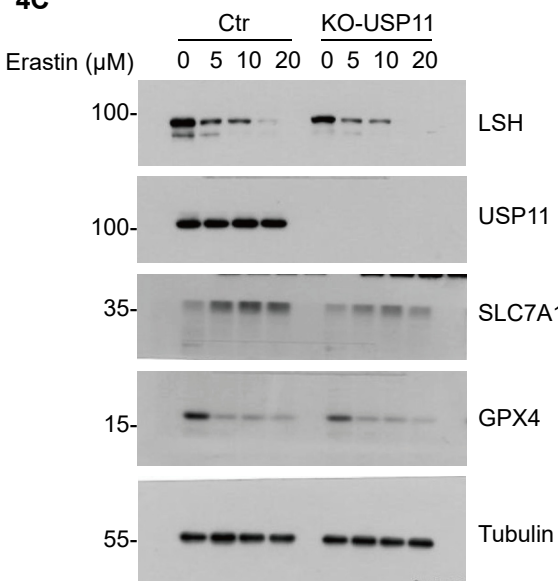

## 4D

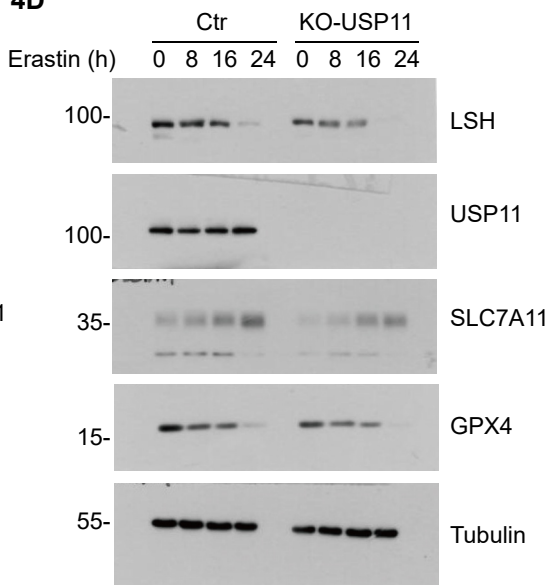

**4E**

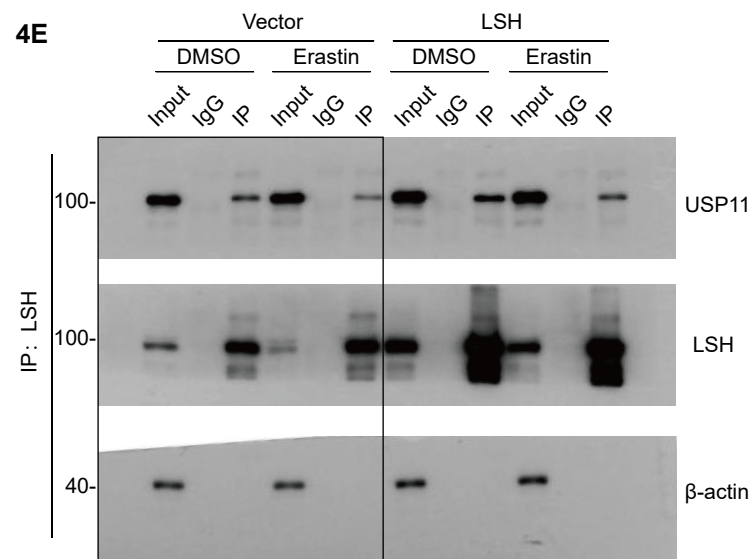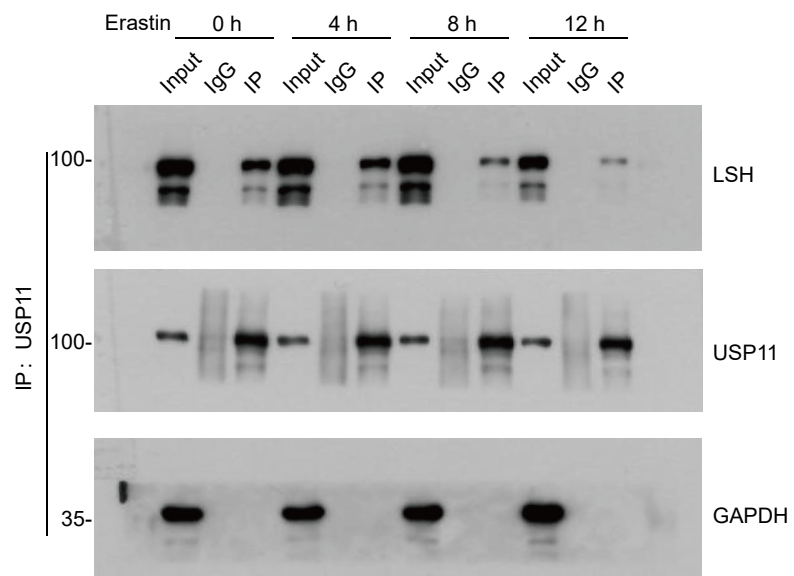

5F

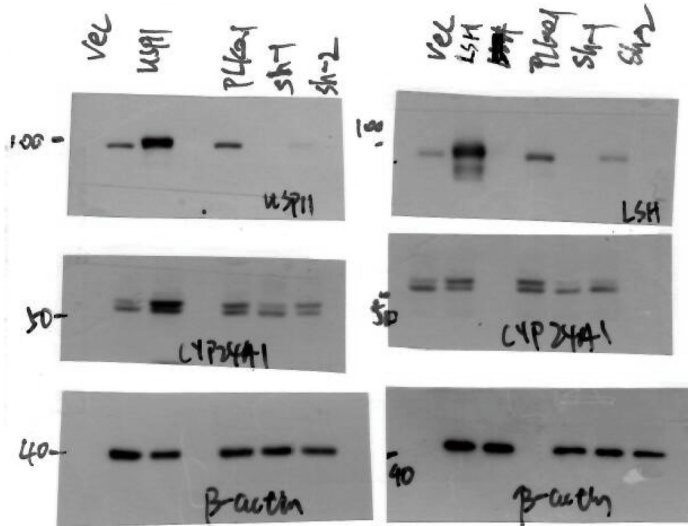

5G

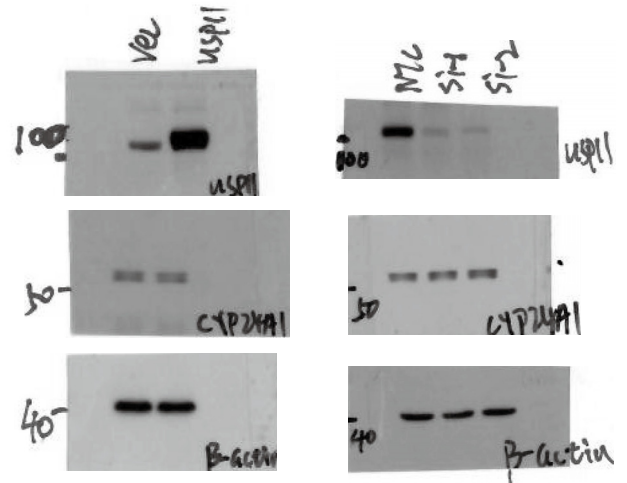

5H

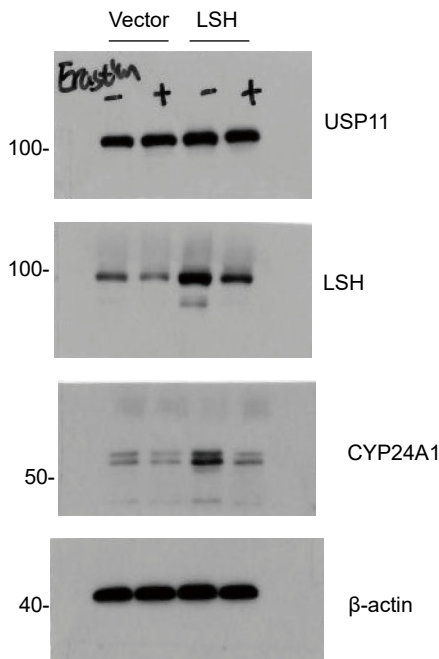

5I

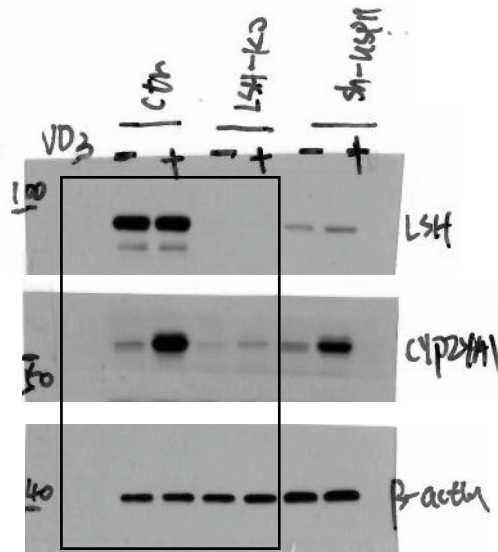

6C

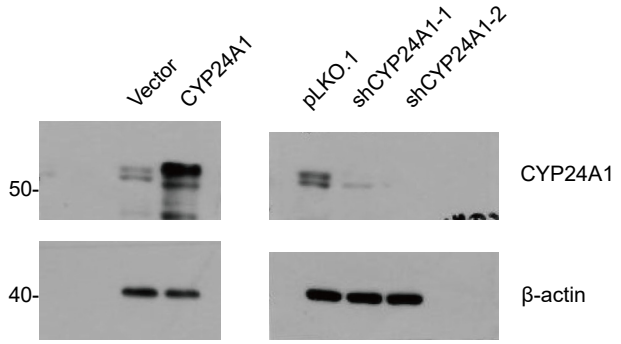

7D

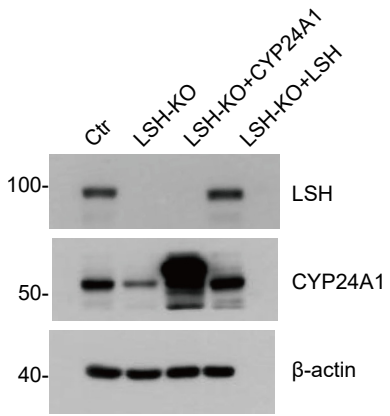

6M

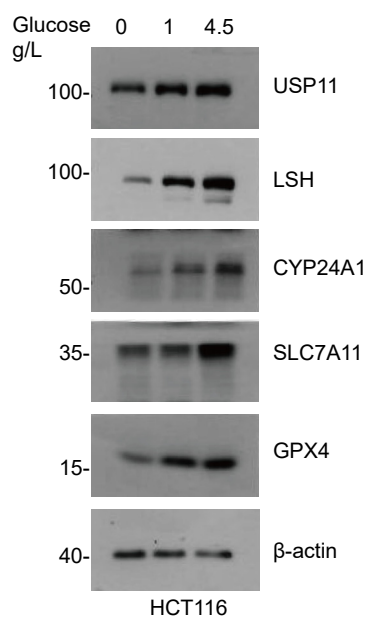

S1A

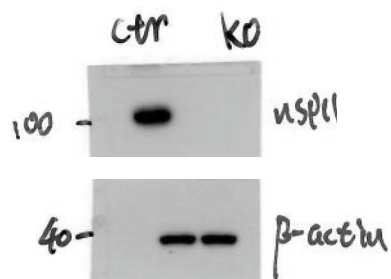

S1B

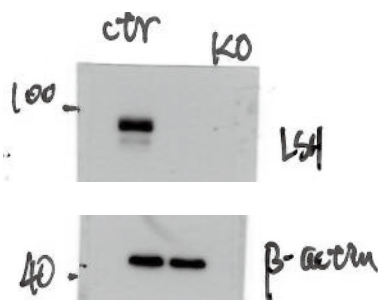

S1C

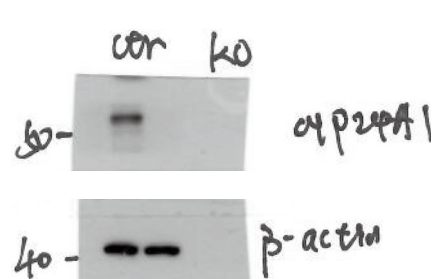

S2B

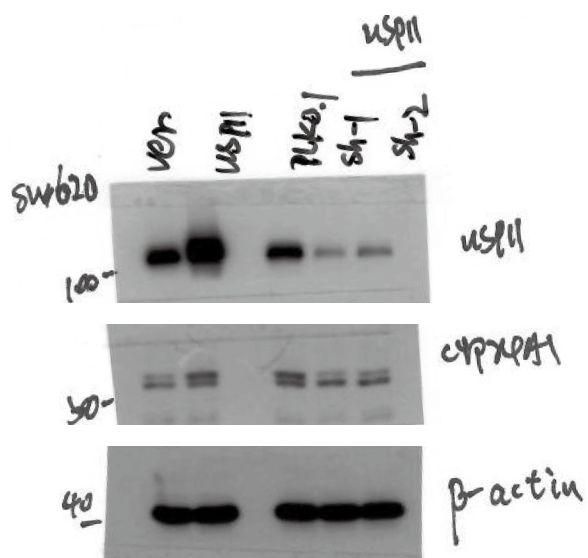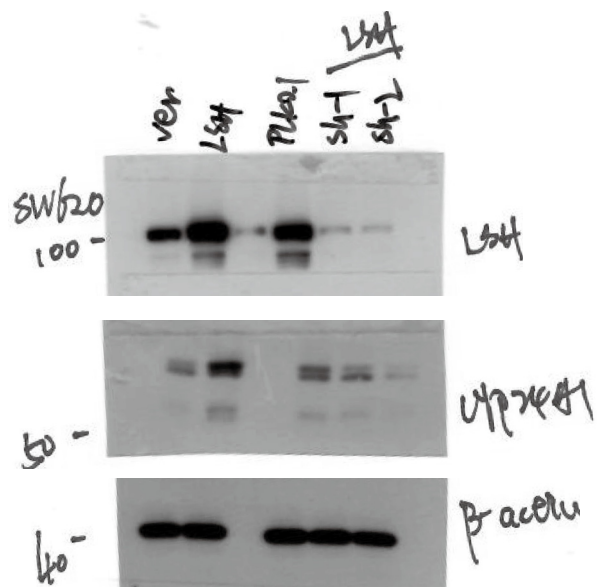

S2C

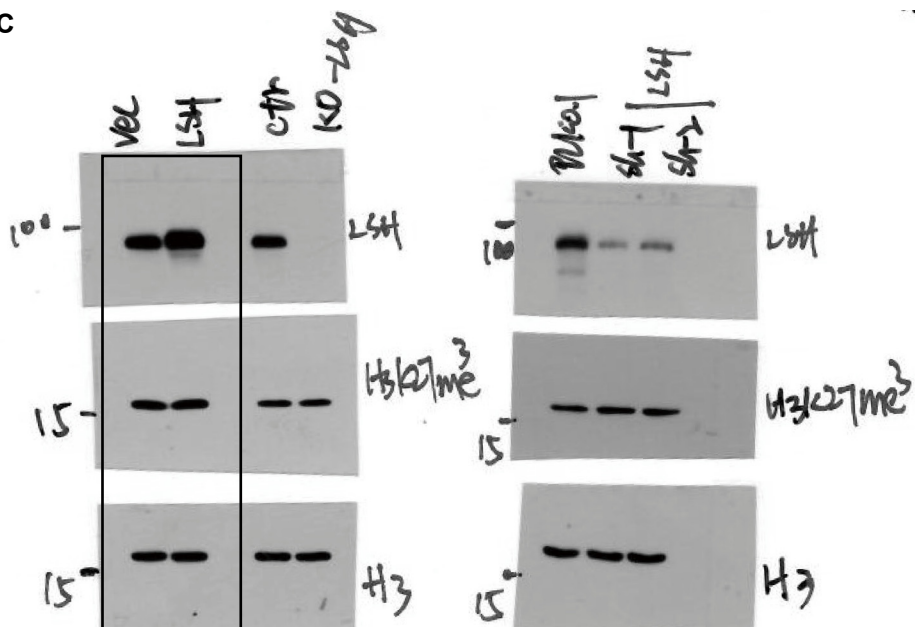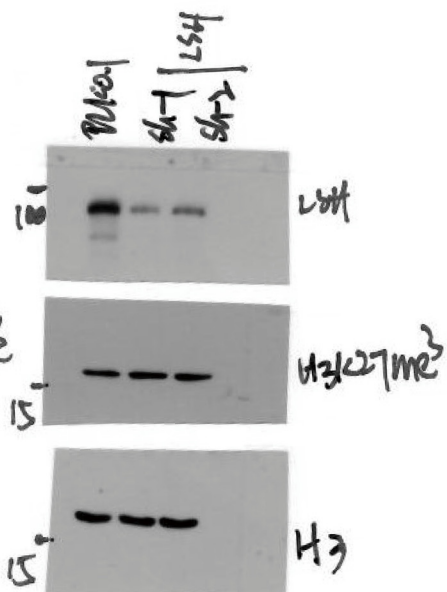

Supplement: Supplementary file 4 — Original western blots [file 41419_2023_5915_MOESM4_ESM.pdf]
